# Artificial sweeteners and risk of incident cardiovascular disease and mortality: evidence from UK Biobank

**DOI:** 10.1186/s12933-024-02333-9

**Published:** 2024-07-04

**Authors:** Tao Sun, Juan Yang, Fang Lei, Xuewei Huang, Weifang Liu, Xingyuan Zhang, Lijin Lin, Linsu Sun, Xinlan Xie, Xiao-Jing Zhang, Jingjing Cai, Zhi-Gang She, Chengsheng Xu, Hongliang Li

**Affiliations:** 1 Department of Cardiology, Renmin Hospital of Wuhan University, 99 Zhangzhidong Rd, 430060 Wuhan, China; 2 Department of Cardiology, Huanggang Central Hospital of Yangtze University, Huanggang, China; 3 State Key Laboratory of New Drug Discovery and Development for Major Diseases, Gannan Medical University, Ganzhou, China; 4 Gannan Innovation and Translational Medicine Research Institute, Gannan Medical University, Ganzhou, China; 5 Institute of Model Animal, Wuhan University, Wuhan, China; 6 Medical Science Research Center, Zhongnan Hospital of Wuhan University, Wuhan, China; 7 Department of Cardiology, The Third Xiangya Hospital, Central South University, Changsha, China; 8 School of Basic Medical Science, Wuhan University, Wuhan, China

## Abstract

**Background:**

Artificial sweeteners are widely popular worldwide as substitutes for sugar or caloric sweeteners, but there are still several important unknowns and controversies regarding their associations with cardiovascular disease (CVD). We aimed to extensively assess the association and subgroup variability between artificial sweeteners and CVD and CVD mortality in the UK Biobank cohort, and further investigate the modification effects of genetic susceptibility and the mediation role of type 2 diabetes mellitus (T2DM).

**Methods:**

This study included 133,285 participants in the UK Biobank who were free of CVD and diabetes at recruitment. Artificial sweetener intake was obtained from repeated 24-hour diet recalls. Cox proportional hazard models were used to estimate HRs. Genetic predisposition was estimated using the polygenic risk score (PRS). Furthermore, time-dependent mediation was performed.

**Results:**

In our study, artificial sweetener intake (each teaspoon increase) was significantly associated with an increased risk of incident overall CVD (HR1.012, 95%CI: 1.008,1.017), coronary artery disease (CAD) (HR: 1.018, 95%CI: 1.001,1.035), peripheral arterial disease (PAD) (HR: 1.035, 95%CI: 1.010,1.061), and marginally significantly associated with heart failure (HF) risk (HR: 1.018, 95%CI: 0.999,1.038). In stratified analyses, non-whites were at greater risk of incident overall CVD from artificial sweetener. People with no obesity (BMI < 30 kg/m^2^) also tended to be at greater risk of incident CVD from artificial sweetener, although the obesity interaction is not significant. Meanwhile, the CVD risk associated with artificial sweeteners is independent of genetic susceptibility, and no significant interaction exists between genetic susceptibility and artificial sweeteners in terms of either additive or multiplicative effects. Furthermore, our study revealed that the relationship between artificial sweetener intake and overall CVD is significantly mediated, in large part, by prior T2DM (proportion of indirect effect: 70.0%). In specific CVD subtypes (CAD, PAD, and HF), the proportion of indirect effects ranges from 68.2 to 79.9%.

**Conclusions:**

Our findings suggest significant or marginally significant associations between artificial sweeteners and CVD and its subtypes (CAD, PAD, and HF). The associations are independent of genetic predisposition and are mediated primarily by T2DM. Therefore, the large-scale application of artificial sweeteners should be prudent, and the responses of individuals with different characteristics to artificial sweeteners should be better characterized to guide consumers’ artificial sweeteners consumption behavior.

**Supplementary Information:**

The online version contains supplementary material available at 10.1186/s12933-024-02333-9.

## Background

Cardiovascular disease (CVD) is the leading cause of mortality and disability, responsible for more than 18 million deaths and 34 million years lived with disability worldwide, as shown in the latest Global Burden of Disease study [1]. Exploring modifiable risk factors for CVD has been a key element in promoting clinical prevention and protecting public health. Added sugar (caloric sweeteners) intake has been recognized as an important risk factor for CVD [2–4], and has been advised to be reduced as far as possible in the whole life cycle of the intake [5]. Thus, artificial sweeteners, as an additive that can reproduce sweetness without the use of sugar, have been widely replacing added sugar in recent years [6, 7]. Nevertheless, some previous studies have reported potential relationships between artificial sweeteners and CVD risk, with mixed evidence [8–21]. It should be noted that those observational perspective studies have mostly used low-calorie beverages or artificial sweetened beverages (ASB) as proxies to explore their effects on CVD risk, without considering the confounding effects of other substances in beverages and tabletop artificial sweeteners (e.g., Canderel) added to the daily diet, which is also a major source of artificial sweetener intake and has simpler ingredients. Recently, the large prospective study from the NutriNet-Santé Cohort provided timely, high-quality evidence on the association of quantitative artificial sweeteners (mg/d) rather than ASB and hard endpoints of CVD (including CHD, cerebrovascular disease) [21], but some important issues remain to be solved. For example, studies on the associations between artificial sweetener intake and some specific CVD subtypes and CVD mortality remain limited or controversial. Meanwhile, given the worldwide popularity of artificial sweetener consumption, the identification of potential high-risk individuals is important because it allows the targeting of preventive measures and guideline development. In addition to non-genetic factors, the potential modification role of genetic susceptibility on the relationship between artificial sweetener intake and CVD risk is also unknown. The interaction between genetics and a modifiable lifestyle has always been a matter of great concern. In June 2023, the statement of the WHO encouraging in-depth conversations before implementing ‘Use of non-sugar sweeteners: WHO guideline’ as policy also highlighted the need for more evidence in this area [22]. Furthermore, as a substitute for added sugar, a potential association between artificial sweeteners and type 2 diabetes mellitus (T2DM) has been widely explored in many previous studies, although the evidence is mixed [23–28]. Based on the fact that diabetes is one of the most important risk factors for cardiovascular disease, it is crucial to explore whether and to what extent T2DM contributes to the effect of artificial sweetener intake on CVD. This is crucial for us to further improve our understanding of underlying mechanisms and to test hypotheses from an epidemiological perspective.

Therefore, we conducted the prospective study using UK Biobank data to extensively explore the association of artificial sweetener intake with CVD (overall, CAD, PAD, stroke, HF, and AF) and CVD mortality. Our study included people who consume tabletop artificial sweeteners (such as Canderel) in their daily diet, which constitutes a representative population engaging in prolonged and intensified consumption of synthetic sweeteners, and excluded individuals with ASB intake avoiding their confounding effect. We also explored the possible variability of the effect of artificial sweeteners on CVD in different populations. Additionally, we further perform a first exploration of the modification effect of genetic susceptibility on associations between artificial sweetener intake and CVD and the potential mediating roles of T2DM.

## Materials and methods

### Data source and study design

The UK Biobank comprises data from a population-based cohort study that recruited more than 500,000 participants (37 to 73 years old). Participants attended one of the 22 assessment centers across England, Scotland, and Wales between 2006 and 2010. Their extensive information on social demography, lifestyle, health, and physical assessments was collected via questionnaires, interviews, health records, physical measures, and blood samples.

**Fig. 1:**
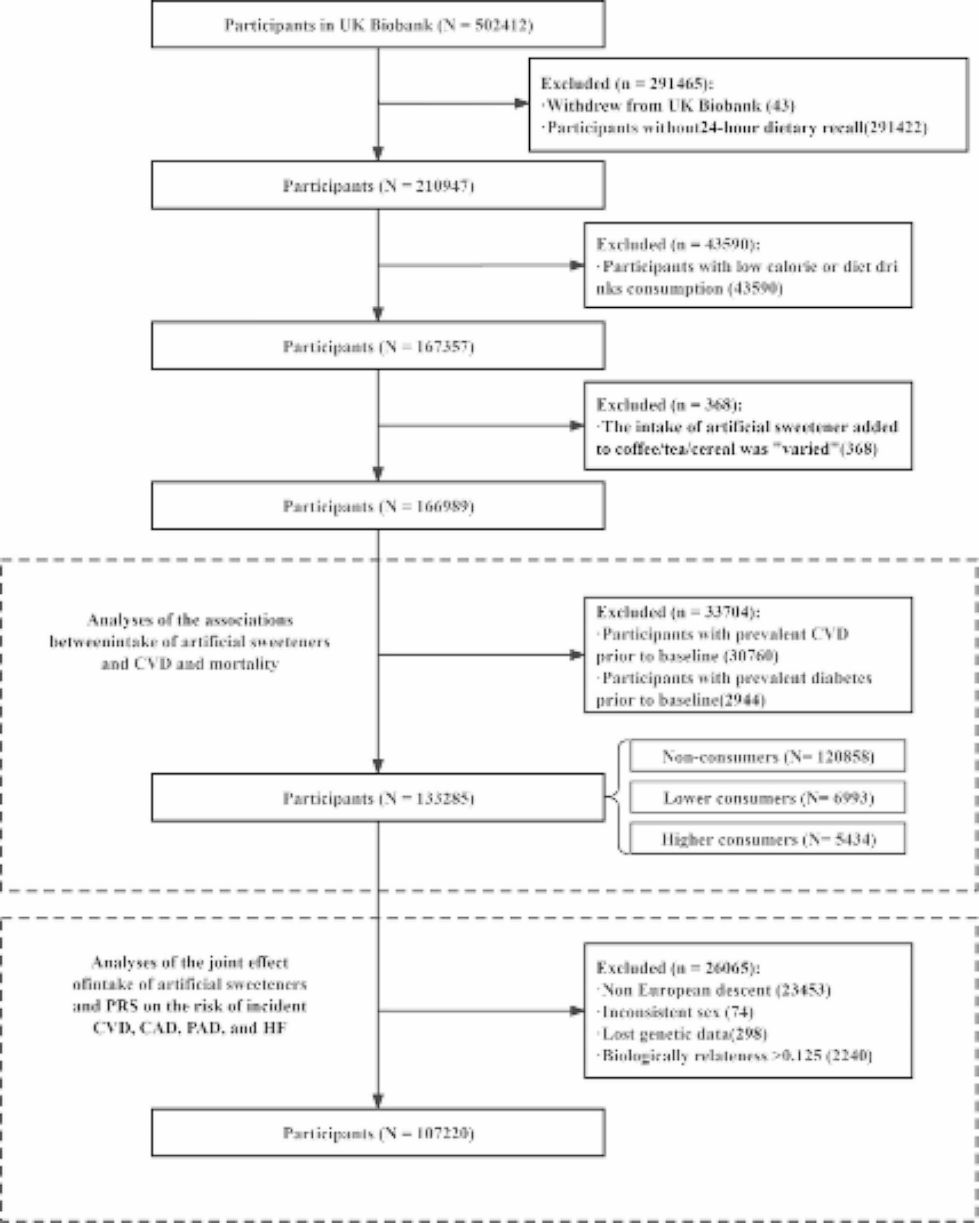
Flow of participants in current UK Biobank study

In the present study, among all 502,412 individuals in the UK Biobank, we first excluded participants who withdrew their data (*n* = 43). Then, we excluded participants with no dietary intake data (the 24-hour recall questionnaire) (*n* = 291,422), which meant that information on artificial sweetener consumption and other dietary information was not available (Table S1). In the remaining 210,947 participants, we extracted all available data for the intake of artificial sweeteners in the 24-hour dietary questionnaire (Table S2). The individuals with data on “Intake of artificial sweetener added to coffee”, “Intake of artificial sweetener added to tea”, and “Intake of artificial sweetener added to cereal” were the population with information on consuming tabletop artificial sweeteners (such as Canderel) and were included in our study. We further excluded individuals as follows: (1) We excluded participants with low-calorie drink intake (*n* = 43,590). The “low-calorie drink intake” in UK Biobank was the ASB (artificial sweetener beverages) consumption used in many previous studies [20, 29], which may contain various additives and are unable to quantify the amounts of artificial sweeteners. (2) Those with an uncertain amount of artificial sweetener intake were also excluded (i.e., individuals who answered “varied” in the intake of added artificial sweeteners in coffee/tea/cereals; *n* = 368). (3) All participants diagnosed with CVD (*n* = 30,760) before baseline were excluded. Participants with prevalent diabetes (*n* = 2,944) (self-reported diagnosis and medication, hospital episode statistics data, and random blood glucose > 11.1 mmol/L) were also excluded. Finally, 133,285 participants were included in the present study. The complete flowchart of participant selection is shown in Fig. 1.

To further explore the associations of artificial sweetener intake and genetics with the risk of CVD, we excluded participants of non-European descent (*n* = 23,453), those with inconsistent sex (*n* = 74), those with missing genetic data (*n* = 298), and those with biologically relatedness > 0.125 (*n* = 2,240). The reason for excluding non-European descent is that the GWAS of CVDs is mainly based on populations of European ancestry. And excluding those with biologically relatedness > 0.125 improves the independence of our samples and strengthens the robustness of our results. Finally, 107,220 participants were included in the analysis.

The UK Biobank received ethical approval from the North West Multicenter Research Ethical Committee. All participants signed written consent. The present study was performed under UK Biobank application number 77,195.

### Assessment of artificial sweetener intake

Dietary information was collected using the Oxford WebQ (www.ceu.ox.ac.uk/research/oxford-webq), a web-based dietary assessment tool recording the consumption of up to 206 widely consumed foods and 32 types of beverages in the previous 24 h [30, 31]. The accuracy of this approach has been considered acceptable, and this approach has been widely used for studying the association between diet and various health outcomes [30, 32, 33]. UK Biobank participants were invited to complete the Oxford WebQ on five occasions over five years, and we calculated mean values from the available data. As we described above, after completely screening all data-fields in the 24-hour dietary questionnaire, the data finally included in our study for artificial sweetener intake were “Intake of artificial sweetener added to coffee”, “Intake of artificial sweetener added to tea”, and “Intake of artificial sweetener added to cereal” (Table S2). Participants were asked, “How many teaspoons/tablets of sweetener (e.g., Canderel) did you add to your coffee (per drink)? /add to your tea/infusion (per drink)? /add to your cereal or porridge (per bowl)?”.

To calculate the intake of artificial sweeteners, we also need to know the specific coffee (cups/mugs), tea (cups/mugs), and cereal (bowls) intake. We further screened the data-fileds in the 24-hour dietary questionnaire, and the total coffee consumption was obtained by summing the number of cups per day of six mutually exclusive coffee types (“Instant coffee intake”, “Filtered coffee intake”, “Cappuccino intake”, “Latte intake”, “Espresso intake”, and “Other coffee type”). The total tea consumption was obtained by summing the cups of five mutually exclusive tea types (“Standard tea intake”, “Rooibos tea intake”, “Green tea intake”, “Herbal tea intake”, and “Other tea intake”). The total cereal consumption was also obtained by summing the bowls of eight mutually exclusive cereal types (“Porridge intake”, “Muesli intake”, “Oat crunch intake”, “Sweetened cereal intake”, “Plain cereal intake”, “Bran cereal intake”, “Whole-wheat cereal intake”, and “Other cereal intake”). Finally, we can calculate the artificial sweetener intake for each participant in our study.

### CVD outcomes and CVD mortality assessment

Cardiovascular outcomes of interest included overall CVD, coronary artery disease (CAD), peripheral arterial disease (PAD), stroke, heart failure (HF), and atrial fibrillation (AF). Through medical record linkages in the UK Biobank, CVD outcomes and CVD mortality were ascertained using the 10th and 9th International Classification of Diseases Revisions. We defined incident overall CVD by ICD-9: 390–459 and ICD-10: I00-I99. Furthermore, definitions of CAD, PAD, stroke, HF, and AF are also provided in Table S3. The date of death and underlying primary cause of death were obtained from death certificates held by the National Health Service (NHS) Information Centre (England and Wales) and the NHS Central Register Scotland (Scotland). Dates and causes of hospital admission were identified via record linkage to Health Episode Statistics (England and Wales) and the Scottish Morbidity Records (SMR01) (Scotland). Details of the linkage procedure can be found at http://content.digital.nhs.uk/services. Person-years were computed by measuring the time from the date of diet questionnaire completion to the date of diagnosis, loss to follow-up, death, or October 31, 2022—whichever came first.

### Polygenic risk score

Detailed information about the genotyping, imputation, and quality control in UK Biobank was reported previously [34]. The polygenic risk score (PRS) of CVD was extracted from ‘Standard PRS (Category 301)’ provided by the UK Biobank PRS Release. Furthermore, to quantify the genetic risk of CAD, PAD, and HF, we obtained the imputed genotype data from UK Biobank, and selected single-nucleotide polymorphisms (SNPs) extracted from published genome-wide association studies, respectively. The construction of the PRS for CAD included 74 SNPs [35, 36], for PAD included 19 SNPs [37], and for HF included 12 SNPs [38], which are listed in Table S4. More details on the SNPs for these CVD subtypes can be found in these published genome-wide association studies. Using a weighted method, PRS = β_1_*SNP_1_ + β_2_*SNP_2_ +… + β_n_*SNP_n_, with each SNP recoded as 0, 1, or 2, according to the number of risk alleles. Furthermore, we categorized the participants into “low genetic risk” and “high genetic risk” groups according to median PRS.

### Covariates

A series of covariates in the present study were collected from touch-screen questionnaires, anthropometric measurements, biochemical indexes, and 24-hour dietary recall questionnaires. The potential confounders include age (continuous), sex (males/females), ethnicity (White/non-White), body-mass index (BMI) (normal/underweight (< 25 kg/m^2^)/overweight (25 ≤ to < 30 kg/m^2^)/obese (≥ 30 kg/m^2^)), systolic blood pressure (SBP) (continuous), low density lipoprotein cholesterol (LDL-C) (continuous), Townsend deprivation index (continuous), cigarette smoking (never/previous/current), frequency of alcohol intake (not current/less than three times a week/three or more times a week), educational level (degree or above/any other qualification/no qualification), physical activity (metabolic equivalent task-min/week (MET-min/week)) (continuous), use of lipid-lowering medication (yes/no), total energy intake (continuous), total sugar intake (continuous), sodium intake(continuous), red and processed meat consumption (continuous), fruit intake (continuous), vegetable intake (continuous), saturated fatty acid intake (continuous), monounsaturated fatty acid intake (continuous), and fibre intake (continuous). The Townsend deprivation index is a composite measure of deprivation based on unemployment, non-car ownership, non-home ownership, and household overcrowding [39]. The small number of missing values for these covariates were imputed by a random forest model based on the rest of the variables in the dataset. More details about the method of imputation were reported in previous studies [40, 41]. The overall missing rate (total missing values among all values) of the covariates is 2.07%. And detailed information on missing rates for each covariate for all participants in the analysis is shown in Table S5.

### Statistical analysis

Baseline characteristics of participants were presented through descriptive statistics and SD for continuous variables and distribution differences (i.e., counts and percentages) for categorical variables. First, we extensively investigated the associations of artificial sweetener intake with incident CVD mortality, CVD, CAD, PAD, stroke, HF, and AF using multivariable Cox proportional hazards models. Regression models were adjusted for age, sex, ethnicity, BMI, SBP, LDL-C, Townsend Deprivation Index, cigarette smoking, alcohol consumption, qualification, physical activity, use of lipid-lowering medication, total energy, total sugars, sodium, red and processed meat, fruit, vegetables, saturated fatty acids, monounsaturated fatty acids, and fibre. Hazard ratios (HRs) and 95% CIs were calculated for each teaspoon increase in artificial sweetener intake. Besides, we assessed the shape of the relationship between artificial sweetener intake and incident cardiovascular disease mortality, cardiovascular disease, coronary artery disease, peripheral arterial disease, stroke, and heart failure using restricted cubic spline (RCS) functions with 3 knots at the 10th, 50th, and 90th percentiles. We also categorized the intake of artificial sweeteners into non-consumers, lower consumers, and higher consumers. The lower consumer and the higher consumer groups were divided by the median value (4 teaspoons/day). The control group was the non-consumers of artificial sweeteners. As categorical variables, we investigated the association between artificial sweeteners and CVD (Table S6). Then, propensity score matching (PSM) was used to match high consumers with non-consumers. The PSM cohorts were identified by balancing all covariates in the cox regression. The matching ratio was 1:3 for higher consumers versus non-consumers. Exact matching with a caliper size of 0.05 was applied for all matching pairs according to the propensity scores. If the estimated standardized difference after matching is less than 0.1, the balance between covariates is considered qualified balancing [42]. The baseline information after matching and the results of cox regression are presented in Tables S7 and S8.

For exploring the effects of artificial sweetener intake on incident overall CVD, CAD, PAD, and HF in different populations and potential variations, subgroup analyses stratified by age (< 65 years old or ≥ 65 years old), sex (males and females), ethnicity (White/non-White), obesity (BMI < 30/≥ 30 kg/m^2^), smoking (never/previous/current), alcohol consumption (not current/less than three times a week/three or more times a week), educational level (degree or above/any other qualification/no qualification), and physical activity (< 600/ 600–1500/ 1500 MET-min/week) were conducted. The levels [low (< 600 MET-min/week), moderate (600–1500 MET-min/week), and high (> 1500 MET-min/week)] of physical activity were defined using standardised IPAQ processing guidelines [43]. To explore the modification role of the PRS on associations between artificial sweetener intake and overall CVD, CAD, PAD, and HF, multiplicative interactions were assessed by comparing multiplicative models with and without an interaction term using the log-likelihood ratio test, and additive interactions were assessed by relative excess risks due to interaction (RERI). The confidence intervals of the RERI and the attributable proportion due to interaction would not contain 0, manifesting additive interaction [44, 45]. Subgroup analyses stratified by genetic risk were also performed. Moreover, to further validate the stability of the results, we also adjusted for the potential confounding of genetic factors by additionally including PRS and the first 10 principal components of ancestry in the main analysis.

We further conducted mediation analyses to determine whether and to what extent the artificial sweetener intake-overall CVD, CAD, PAD, and HF relationships are mediated by prior T2DM. Specifically, a logistic regression model was first constructed with artificial sweetener intake (exposure) as the independent variable and incident T2DM (mediator) during follow-up as the dependent variable, and then a cox proportional hazards model was fitted with the aforementioned exposure and mediation as the independent variables and event CVD (or CAD, PAD, or HF) as the dependent variable. Finally, the direct effect (DE), indirect effect (IE), and total effect (TE) were calculated from the two regressions [46, 47].

Sensitivity analyses were conducted in four ways (based on the main model): (1) We conducted the main analyses after excluding events that took place within two years of follow-up to reduce the potential influence of reverse causality. (2) We conducted the main analyses among those completing two or more 24-hour dietary questionnaires. (3) We conducted the main analyses among participants with complete covariate data. (4) We conducted the main analyses within the population, which included individuals with baseline diabetes, and further adjusted for this factor. (5) We further adjusted the consumption clusters (including adjusting for ‘added sugars and preserves’ instead of total sugar intake in the main model, or further adjusting for coffee consumption, tea consumption, and cereal consumption, or further adjusting for “ideal diet” (yes/no) instead of red and processed meat, fruit, and vegetables). The details of the “ideal diet” were shown in our previous study [48].

In our analyses, there was no violation of the proportionality assumption by calculating the Schoenfeld residuals and plotting the scaled Schoenfeld residuals against time. Meanwhile, no multicollinearity problem was found when we examined collinearity between all covariates included in these analyses by correlation matrix analysis. All statistical analyses were carried out using the R software (version 4.2.0). P values < 0.05 were considered statistically significant.

## Result

### Baseline characteristics

The baseline information of 133,285 participants involved in the analysis of the associations between artificial sweetener intake and incident CVD and CVD mortality is shown in Table 1. The mean age was 55.7 (SD, 7.9) years, and 43.7% of participants were male. Compared with the participants who did not consume artificial sweeteners, the participants with higher artificial sweetener intake were more likely to be older, male, white, obese, smokers, less educated, at higher levels of material deprivation, have a higher baseline SBP, have more physical activity (MET-min/week), have more use of lipid-lowering medication, and were more likely to intake more sodium, more red and processed meat, and less fruit, vegetables, and fibre.

**Table 1:** Baseline characteristics of the study population

Characteristics	Overall (*n* = 133,285)	Artificial sweeteners			*P* value
		Non-consumers (*n* = 120,858)	Lower consumers (*n* = 6993)	Higher consumers (*n* = 5434)	
Age, (years, mean (SD))	55.7 (7.9)	55.6 (7.9)	56.6 (7.9)	57.4 (7.6)	< 0.001
**Sex, n (%)**					< 0.001
Female	75,060 (56.3)	67,910 (56.2)	4284 (61.3)	2866 (52.7)	–
Male	58,225 (43.7)	52,948 (43.8)	2709 (38.7)	2568 (47.3)	–
**Ethnicity**					< 0.001
Non-White	6205 (4.7)	5618 (4.7)	392 (5.6)	195 (3.6)	
White	127,020 (95.3)	115,184 (95.3)	6597 (94.4)	5239 (96.4)	
BMI (kg/m^2^, mean (SD))	26.2 (4.2)	26.1 (4.1)	27.5 (4.5)	27.9 (4.6)	< 0.001
**BMI category (kg/m2), n (%)**					< 0.001
< 25	57,577 (43.2)	53,976 (44.7)	2152 (30.8)	1449 (26.7)	–
25–30	54,587 (41.0)	48,818 (40.4)	3229 (46.2)	2540 (46.7)	–
≥ 30	21,121 (15.8)	18,064 (14.9)	1612 (23.1)	1445 (26.6)	–
SBP (mmHg, mean (SD))	135.8 (18.2)	135.6 (18.2)	136.9 (18.1)	138.2 (18.1)	< 0.001
LDL-C (mmol/L, mean (SD))	3.6 (0.8)	3.6 (0.8)	3.6 (0.9)	3.6 (0.9)	0.645
Townsend deprivation index (mean (SD))	− 1.6 (2.9)	− 1.6 (2.9)	− 1.6 (2.9)	− 1.5 (2.9)	0.002
MET (minutes/week, mean (SD))	974.9 (1013.3)	971.7 (1008.7)	1,005.7 (1052.0)	1,008.2 (1063.2)	0.004
**MET category (minutes/week), n (%)**					< 0.001
< 600	56,408 (42.3)	51,239 (42.4)	2924 (41.8)	2245 (41.3)	–
600–1500	57,517 (43.2)	52,269 (43.2)	2977 (42.6)	2271 (41.8)	–
≥ 1500	19,360 (14.5)	17,350 (14.4)	1092 (15.6)	918 (16.9)	–
**Cigarette smoking, n (%)**					< 0.001
Never	77,883 (58.6)	71,915 (59.6)	3660 (52.5)	2308 (42.6)	–
Previous	44,560 (33.5)	39,476 (32.7)	2737 (39.3)	2347 (43.3)	–
Current	10,528 (7.9)	9188 (7.6)	574 (8.2)	766 (14.1)	–
**Alcohol consumption, n (%)**					< 0.001
Not current	7570 (5.7)	6694 (5.5)	462 (6.6)	414 (7.6)	–
Two or fewer times a week	59,408 (44.6)	53,094 (44.0)	3467 (49.6)	2847 (52.4)	–
Three or more times a week	66,195 (49.7)	60,968 (50.5)	3055 (43.7)	2172 (40.0)	–
**Education level, n (%)**					< 0.001
No qualification	9792 (7.4)	8294 (6.9)	741 (10.7)	757 (14.0)	–
Any other qualification	62,473 (47.1)	55,471 (46.1)	3877 (55.8)	3125 (57.8)	–
Degree or above	60,413 (45.5)	56,549 (47.0)	2336 (33.6)	1528 (28.2)	–
**Use of lipid-lowering medication, n (%)**					< 0.001
No	120,645 (90.9)	110,068 (91.5)	6026 (86.6)	4551 (84.2)	–
Yes	12,040 (9.1)	10,251 (8.5)	935 (13.4)	854 (15.8)	–
Total energy (kj, mean (SD))	8648.2 (2511.3)	8668.9 (2506.3)	8313.8 (2514.7)	8617.9 (2586.4)	< 0.001
Total sugars (mean (SD))	125.3 (49.1)	125.4 (49.0)	122.8 (50.0)	125.2 (51.2)	< 0.001
Sodium (mg, mean (SD))	1935.3 (783.7)	1933.0 (782.9)	1904.0 (763.7)	2025.2 (820.2)	< 0.001
Red and processed meat (mean (SD))	12.6 (2.9)	12.6 (3.0)	12.8 (2.6)	13.0 (2.6)	< 0.001
Fruit (pieces/day, mean (SD))	2.5 (1.6)	2.5 (1.7)	2.5 (1.5)	2.3 (1.6)	< 0.001
Vegetable (tablespoons/day, mean (SD))	5.1 (3.2)	5.1 (3.2)	5.1 (3.2)	4.9 (3.3)	0.002
Saturated fatty acids (g, mean (SD))	27.3 (12.3)	27.4 (12.3)	25.3 (11.8)	26.9 (12.4)	< 0.001
Monounsaturated fatty acids (g, mean (SD))	26.6 (11.2)	26.7 (11.2)	25.0 (10.8)	26.3 (11.4)	< 0.001
Fibre (g, mean (SD))	17.9 (6.8)	18.0 (6.8)	17.3 (6.8)	17.3 (6.7)	< 0.001
Added sugars and preserves intake (g/day, mean (SD))	9.5 (14.3)	9.6 (14.4)	8.1 (11.8)	9.4 (14.5)	< 0.001
Artificial sweetener intake (teaspoons, mean (SD))	0.4 (1.8)	0.0 (0.0)	2.1 (1.2)	7.8 (3.5)	< 0.001

Townsend deprivation index = a composite area-level measure of deprivation based on unemployment, non-car ownership, non-home ownership, and household overcrowding; a higher score indicates higher deprivation

BMI, body mass index; LDL-C, low density lipoprotein cholesterol; MET, Metabolic Equivalent Task; SBP, systolic blood pressure; SD, standard deviation

Additionally, in Table S1, we found that compared to those who did not respond to the questionnaire, those who responded to the questionnaire were more likely to be younger, female, white, have a lower baseline BMI, fewer smokers, a higher education level, lower levels of material deprivation, a lower baseline SBP, have less physical activity (MET-min/week), and use less lipid-lowering medication.

### Associations of artificial sweetener intake with incident CVD and CVD mortality

Artificial sweetener intake (each teaspoon increase) was significantly associated with an increased risk of incident overall CVD, CAD, PAD, and marginally significantly associated with HF. Table 2 shows the associations between artificial sweetener intake and the risk of incident CVD and CVD mortality in Cox proportional-hazards models. In crude models, we observed that the artificial sweetener intake was significantly associated with an increased risk of incident CVD mortality, overall CVD, CAD, PAD, and HF, except for stroke and AF. After fully adjusting for age, sex, ethnicity, BMI, SBP, LDL-C, Townsend Deprivation Index, cigarette smoking, alcohol consumption, qualification, physical activity, use of lipid-lowering medication, total energy, total sugars, sodium, red and processed meat, fruit, vegetables, saturated fatty acids, monounsaturated fatty acids, and fibre, the artificial sweetener intake was significantly or marginally significantly associated with an increased risk of incident overall CVD, CAD, PAD, and HF. The HR of overall CVD, CAD, PAD, and HF were 1.012 (95%CI: 1.008, 1.017), 1.018 (95%CI: 1.001, 1.035), 1.035 (95%CI: 1.010, 1.061), and 1.018 (95%CI: 0.999, 1.038) for each teaspoon increase in the artificial sweetener intake, respectively (Table 2). Consistent with the result of Cox proportional-hazards models, monotonic linear exposure-response relationships of artificial sweetener intake with risk of incident overall CVD and PAD were also observed in RCS models (Figure S1). The risk of CAD also significantly increases with increasing artificial sweetener intake (overall *P* = 0.005; *P* for non-linear: 0.037). After PSM, the higher consumers also had significantly higher CVD, CAD, PAD, and HF risks when compared with non-consumers (Table S7, Table S8). Subgroup analyses in Table 3 and Table S9-11 revealed that non-whites had a higher estimated hazard of incident overall CVD in association with artificial sweetener intake. Meanwhile, it is worth noting that despite the nonsignificant p values for obesity interaction, the hazard risk for overall CVD tended to be higher for subjects with no obesity (BMI < 30 kg/m^2^) (Obesity: HR: 1.010, 95%CI: 1.002,1.019; non-Obesity: 1.014, 95%CI: 1.008, 1.020; interaction *P*: 0.069).

**Table 2:** Associations between intake of artificial sweeteners (each teaspoon increase) and cardiovascular disease mortality, cardiovascular disease, coronary artery disease, peripheral arterial disease, stroke, heart failure, and atrial fibrillation, with UK Biobank cohort

	Total No. of participants	Cases/person-years	Crude	Model l	Model 2
			HR (95% CI); *P*	HR (95% CI); *P*	HR (95% CI); *P*
CVD mortality	133,285	991/1,457,448	1.037 (1.010, 1.065); 0.007	1.016 (0.989, 1.044); 0.253	1.014 (0.987, 1.043); 0.305
Overall CVD	133,285	38,260/1,250,882	1.033 (1.028, 1.038); < 0.001	1.013 (1.008, 1.018); < 0.001	1.012 (1.008, 1.017); < 0.001
CAD	133,285	2655/1,445,201	1.042 (1.026, 1.059); < 0.001	1.019 (1.002, 1.036); 0.027	1.018 (1.001, 1.035); 0.034
PAD	133,285	857/1,454,570	1.073 (1.048, 1.099); < 0.001	1.037 (1.011, 1.063); 0.004	1.035 (1.010, 1.061); 0.006
Stroke	133,285	1912/1,450,456	1.010 (0.988, 1.033); 0.384	0.999 (0.976, 1.022); 0.925	0.998 (0.975, 1.021); 0.868
HF	133,285	1949/1,451,274	1.045 (1.026, 1.064); < 0.001	1.019 (1.000, 1.039); 0.047	1.018 (0.999, 1.038); 0.061
AF	133,285	5889/1,432,914	1.011 (0.998, 1.024); 0.087	0.999 (0.987, 1.012); 0.914	0.998 (0.986, 1.011);
					0.805

Analyses were conducted using Cox proportional hazard models. Data are hazard ratios (95% CIs). Crude was adjusted for age, sex, ethnicity. Model 1 was adjusted as in Crude and for BMI, SBP, LDL-C, Townsend Deprivation Index, cigarette smoking, alcohol consumption, qualification, physical activity, and use of lipid-lowering medication. Model 2 was adjusted as in model 1 and for total energy, total sugars, sodium, red and processed meat, fruit, vegetables, saturated fatty acids, monounsaturated fatty acids, and fibre. *P* < 0.05 was considered statistically significant

AF, atrial fibrillation; BMI, body mass index; CAD, coronary artery disease; CVD, cardiovascular disease; CI, confidence interval; HF, heart failure; HR, hazard ratio; LDL-C, low density lipoprotein cholesterol; PAD, peripheral arterial disease; SBP, systolic blood pressure

**Table 3:** Stratified analysis of the association between intake of artificial sweeteners (each teaspoon increase) and overall cardiovascular disease incidence

Subgroup	Total no. of participants	Cases/person-years	HR (95% CI)	*P* for interaction
**Age at baseline, years**				
≥ 65	19,575	9618/158,035	1.013 (1.003,1.022)	0.252
< 65	113,710	28,642/1,092,847	1.013 (1.007,1.019)	
**Sex**				
Men	58,225	19,605/528,069	1.011 (1.004,1.017)	0.144
Women	75,060	18,655/722,814	1.015 (1.008,1.023)	
**Ethnicity**				
White	127,080	36,481/1,190,717	1.011 (1.006,1.016)	0.028
Others	6205	1779/60,166	1.042 (1.017,1.068)	
Obesity, kg/m2				
BMI ≥ 30	21,123	8697/182,910	1.010 (1.002,1.019)	0.069
BMI < 30	112,162	29,563/1,067,972	1.014 (1.008,1.020)	
**Smoking status**				
Never	78,130	20,157/748,553	1.010 (1.002,1.019)	0.690
Previous	44,624	14,773/404,499	1.013 (1.006,1.020)	
Current	10,531	3330/97,831	1.014 (1.002,1.026)	
**Alcohol consumption**				
Not current	7570	2405/69,801	0.999 (0.982,1.016)	0.040
Two or fewer times a week	59,478	16,618/563,126	1.018 (1.011,1.025)	
Three or more times a week	66,237	19,237/617,955	1.008 (1.000,1.015)	
**Qualification**				
No qualification	9794	4241/84,386	1.009 (0.998,1.021)	0.742
Any other qualification	62,885	18,916/589,581	1.013 (1.006,1.019)	
Degree or above	60,606	15,103/576,915	1.014 (1.004,1.024)	
**Physical activity, minutes/week**				
MET: <600	56,414	16,014/528,146	1.014 (1.006,1.022)	0.746
MET: 600–1500	57,505	16,667/539,610	1.010 (1.002,1.018)	
MET: ≥1500	19,366	5579/183,126	1.013 (1.002,1.024)	

Data are hazard ratios (95% CIs). Models were adjusted for age, sex, ethnicity, BMI, SBP, LDL-C, Townsend Deprivation Index, cigarette smoking, alcohol consumption, qualification, physical activity, use of lipid-lowering medication, total energy, total sugars, sodium, red and processed meat, fruit, vegetables, saturated fatty acids, monounsaturated fatty acids, and fibre. *P* < 0.05 was considered statistically significant

BMI, body mass index; LDL-C, low density lipoprotein cholesterol; CI, confidence interval; HR, hazard ratio; MET, metabolic equivalent task; SBP, systolic blood pressure

### Associations of artificial sweetener intake and PRS with cardiovascular outcomes

The CVD risk associated with artificial sweeteners is independent of genetic susceptibility, and no significant interaction exists between genetic susceptibility and artificial sweeteners. For the constructed CAD, PAD, and HF PRS, we observed that participants with high genetic risk according to PRS had a higher risk of incident CAD (HR: 1.587, 95%CI: 1.455,1.730), PAD (HR: 1.172, 95%CI: 1.012,1.358), and HF (HR: 1.211, 95%CI: 1.098,1.335) than those with low genetic risk (Table S12-14). Associations between artificial sweetener intake and overall CVD, CAD, PAD, and HF incidence stratified by low and high PRS are presented in Table 4. There was no evidence of significant additive interactions between artificial sweetener intake and PRS on the risk of incident overall CVD (RERI: 0.010, 95% CI:-0.002,0.022), CAD (RERI: 0.007, 95% CI: -0.040,0.053), PAD (RERI: -0.020, 95% CI: -0.082,0.042), and HF (RERI: 0.007, 95% CI: -0.037,0.052). And the multiplicative interaction analyses also did not disclose any significant interactions (Table 4).

**Table 4:** Associations between intake of artificial sweeteners (each teaspoon increase) and coronary artery disease, peripheral arterial disease, and heart failure according to low and high polygenic risk score for each disease in participants from the UK Biobank cohort

	Total No. of Participants	Cases/Person-Years	HR (95% CI)	RERI	*P* for interaction
**CVD PRS**					
Main analysis*	107,220	31,036/1,003,584	1.010 (1.005,1.016)	−	−
Low	53,610	14,326/508,896	1.006 (0.998,1.014)	0.010 ( − 0.002,0.022)	0.169
High	53,610	16,710/494,688	1.014 (1.007,1.022)		
**CAD PRS**					
Main analysis*	107,220	2191/1,160,192	1.020 (1.002,1.039)	−	−
Low	53,610	838/581,463	1.021 (0.993,1.051)	0.007 ( − 0.040,0.053)	0.829
High	53,610	1353/578,729	1.019 (0.996,1.044)		
**PAD PRS**					
Main analysis*	107,220	716/1,167,812	1.024 (0.996,1.053)	−	−
Low	53,608	330/583,780	1.039 (1.001,1.079)	− 0.020 ( − 0.082,0.042)	0.437
High	53,612	386/584,033	1.008 (0.967,1.051)		
**HF PRS**					
Main analysis*	107,220	1629/1,164,989	1.017 (0.996,1.038)	−	−
Low	53,610	740/582,845	1.015 (0.985,1.047)	0.007 ( − 0.037,0.052)	0.873
High	53,610	889/582,144	1.019 (0.991,1.047)		

Analyses were conducted using Cox proportional hazard models. Data are hazard ratios (95% CIs). Models were adjusted for age, sex, BMI, SBP, LDL-C, Townsend Deprivation Index, cigarette smoking, alcohol consumption, qualification, physical activity, use of lipid-lowering medication, total energy, total sugars, sodium, red and processed meat, fruit, vegetables, saturated fatty acids, monounsaturated fatty acids, fibre, and first 10 principal components of ancestry. *P* < 0.05 was considered statistically significant

Main analysis*: On the basis of the main analysis adjusted for age, sex, BMI, SBP, LDL-C, Townsend Deprivation Index, cigarette smoking, alcohol consumption, qualification, physical activity, use of lipid-lowering medication, total energy, total sugars, sodium, red and processed meat, fruit, vegetables, saturated fatty acids, monounsaturated fatty acids, fibre, further adjusted for PRS (category) and first 10 principal components of ancestry

BMI, body mass index; CAD, coronary artery disease; CI, confidence interval; HF, heart failure; HR, hazard ratio; LDL-C, low density lipoprotein cholesterol; PAD, peripheral arterial disease; RERI, relative excess risks due to interaction; SBP, systolic blood pressure

### Mediation analyses of T2DM on associations of artificial sweetener intake with cardiovascular outcomes

**Fig. 2:**
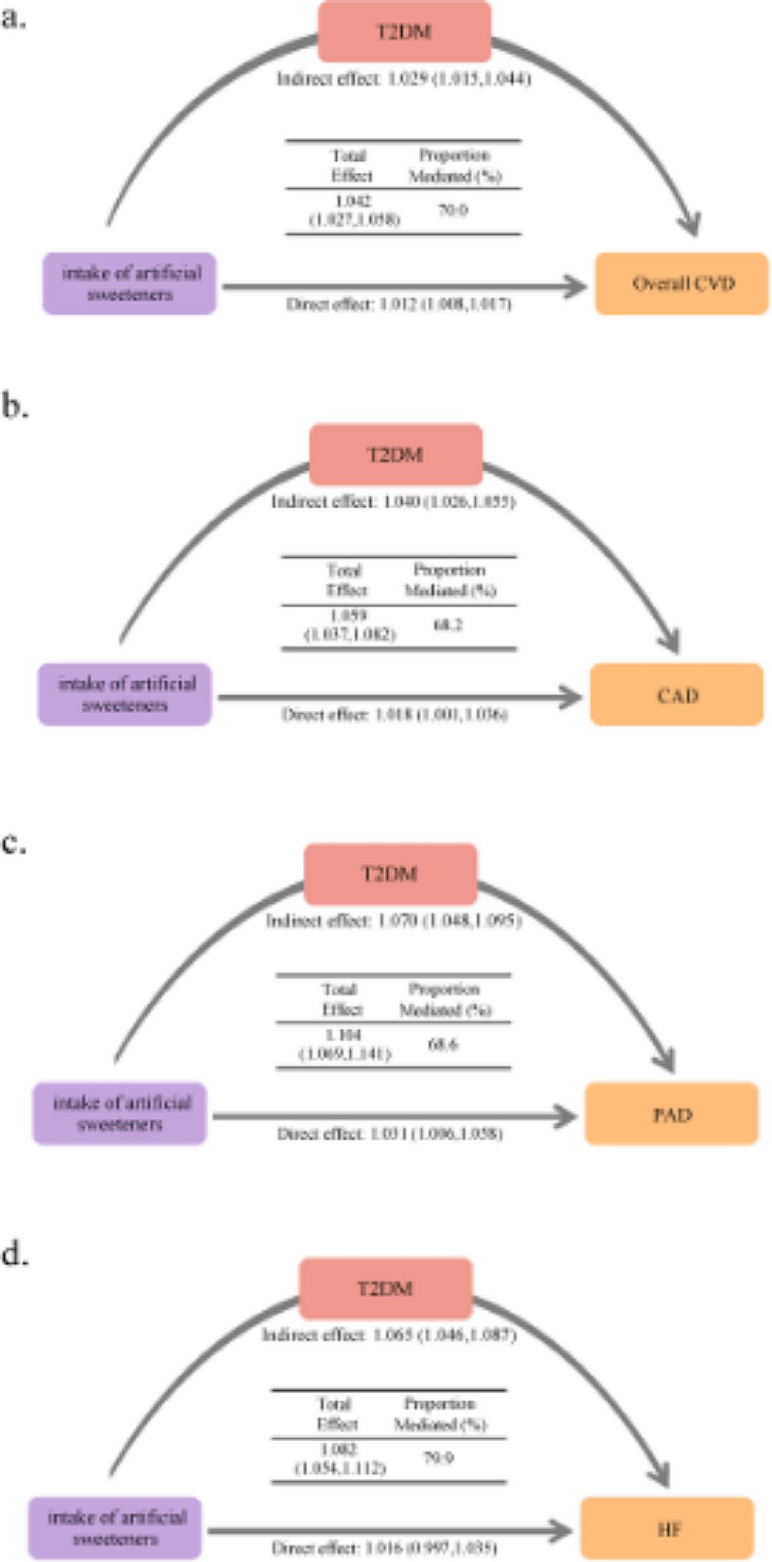
Mediation effect [hazard ratio (95% CI)] of prior T2DM on the association between artificial sweetener intake and CVD (overall CVD, CAD, PAD, and HF). Models were adjusted for age, sex, ethnicity, BMI, SBP, LDL-C, Townsend Deprivation Index, cigarette smoking, alcohol consumption, qualification, physical activity, use of lipid-lowering medication, total energy, total sugars, sodium, red and processed meat, fruit, vegetables, saturated fatty acids, monounsaturated fatty acids, and fibre. BMI, body mass index; CAD, coronary artery disease; CVD, cardiovascular disease; CI, confidence interval; HF, heart failure; HR, hazard ratio; LDL-C, low density lipoprotein; PAD, peripheral arterial disease; SBP, systolic blood pressure

There are significant mediation effects of intermediate diseases (T2DM) on the association between artificial sweetener intake and overall CVD, CAD, PAD, and HF by time-dependent mediation analyses (Fig. 2). We observed that the association of artificial sweetener intake with incident overall CVD was significantly mediated by T2DM, and the IE effect was 1.029 (95% CI: 1.015,1.044), with a proportion mediated of 70.0% (Fig. 2). The associations of artificial sweetener intake with incident CAD, PAD, and HF were also significantly mediated by T2DM, and the IE effect was 1.040 (1.026,1.055), 1.070 (1.048,1.095), and 1.065 (1.046,1.087), with a proportion mediated of 68.2%, 68.6%, and 79.9% (Fig. 2).

### Sensitivity analyses

Several additional sensitivity analyses were performed to evaluate the robustness of our findings, and the results are presented in Table S15-19. These included excluding events that occurred in the first two years of follow-up, restricting the analysis to participants with two or more 24-hour dietary recall questionnaires, restricting the analysis to participants with complete covariate data, not excluding participants with baseline diabetes, and further adjusting for other potential dietary confounders. The results of all sensitivity analyses were generally consistent with the main conclusion.

## Discussion

This perspective study provided important information on the association between artificial sweetener intake and CVD. (1) We found that artificial sweetener intake was associated with an increased risk of incident overall CVD, CAD, PAD, and HF. (2) We further explored the possible variability of the effect of artificial sweeteners on CVD in different populations. Our stratified analyses suggested that non-whites had a higher estimated hazard of incident overall CVD in association with artificial sweetener intake. (3) Our results demonstrate the risk of artificial sweetener intake for incident CVD independent of genetic susceptibility, and there was no evidence of interactions between artificial sweetener intake and genetic susceptibility, whether additive or multiplicative. (4) We found that the relationship between artificial sweetener intake and overall CVD is significantly mediated in large part by prior T2DM (proportion of IE: 70.0%). In specific CVD subtypes (CAD, PAD, and HF), the proportion of IE ranges from 68.2 to 79.9%.

While some studies of ASB and CVD have implied possible associations between artificial sweeteners and CVD [14, 15, 18, 19], there is only one other large-scale prospective study from the NutriNet-Sante cohort that has specifically examined the association between artificial sweetener intake and CVD [21]. In line with the results from the NutriNet-Sante cohort, our study, based on large-scale population data from the UK Biobank, confirms the significant association between artificial sweeteners rather than ASB and overall CVD. Beyond examining the overall risk of CVD, our study further extensively explored the associations and subgroup variances between artificial sweeteners and various specific subtypes of CVD (including CAD, stroke, PAD, HF, and AF).

Our results showed a significant association between artificial sweeteners and CAD, but no significant correlation with stroke. In the prospective NutriNet-Santé cohort, the risk of incident CHD was found to be linked with some specific types of artificial sweeteners (e.g., acesulfame potassium and sucralose), rather than total artificial sweetener consumption [21]. Additionally, although the Nutrine-Sante cohort showed a significant association between artificial sweeteners and cerebrovascular events and transient ischemic events, it also did not find a significant association between artificial sweeteners and stroke. Meanwhile, the magnitude and direction of HR values for stroke of different artificial sweeteners were also various (aspartame: HR:1.25, 95%CI: 0.97,1.59; acesulfame potassium: HR:0.96, 95%CI: 0.59,1.56; sucralose: HR:0.69, 95%CI:0.37,1.29). Therefore, compared with the results of the NutriNet-Santé cohort, our results are generally consistent with them, but there are some differences to some extent. We believed that differences in the types of artificial sweeteners were one of the key factors contributing to the variations in research findings. As mentioned in previous studies, different artificial sweeteners have different biological metabolic effects [21, 49]. For example, aspartame is broken down into phenylalanine, aspartic acid, and methanol, which are further metabolized, whereas potassium acesulfame is absorbed from the small intestine, enters the bloodstream and tissues through circulation, and is then excreted in the urine [49]. In addition, there are also some studies investigating the relationship between ASB and CAD as well as stroke. In line with our study, the Women’s Health Initiative Observational Study (WHI-OS) cohort [16] and the meta-analysis by Jiawei Yin et al. [50] showed an association between ASB and CHD. However, the results of the Nurses’ Health Study (NHS) cohort [51] (< 0/month vs. ≥ 2 ASBs/day; RR: 1.15; 95% CI: 0.97, 1.38; *P* = 0.07 and the WHO meta-analyses [27] which included four prospective cohort studies, only showed a nonsignificant increase (HR:1.16, 95%CI: 0.97,1.39). Regarding stroke, studies such as the Nurses’ Health Study [10], the Women’s Health Initiative Observational Study [16], the community-based Framingham Heart Study Offspring cohort [52] and meta-analyses [19, 27] showed a significant association between ASB and stroke, but conflicting results also exist [9, 17].

At present, the evidence of associations between PAD, HF, AF, and artificial sweeteners is very limited. The only two studies that have examined the association between ASB and HF have controversial conclusions [18, 20]. The Women’s Health Initiative (WHI) cohort suggested that ASB was not significantly associated with HF (< 1 serving/week vs. ≥ 1 serving/day; HR: 0.96, 95%CI: 0.72, 1.28) [18]. In contrast, the results of a prospective cohort study from UK Biobank showed that individuals who consumed > 2 L/week ASBs had an increased risk of HF (HR: 1.30, 95%CI: 1.16–1.47) [20]. There are still some gaps in studies using ASB as a proxy, such as not considering other major sources of artificial sweetener intake and the possible confounding of various additives in ASB. Our study first demonstrated the significant associations of artificial sweetener intake rather than ASB with PAD and the marginally significant associations of artificial sweetener intake with HF, providing important evidence for the risk of HF and PAD with artificial sweeteners. At the same time, in our study, we broadly excluded all patients with CVD (I00-I99) or diabetes patients at baseline, which may represent a relatively healthier population. This also indicates that even among relatively healthy individuals without pre-existing cardiovascular disease or diabetes, the consumption of artificial sweeteners is still linked to an elevated risk of CVD. This further emphasizes the need for caution regarding the public consumption of artificial sweeteners. Similarly, for these cardiovascular subgroups, appropriately designed and large sample studies are needed in the future with more detailed analyses of specific artificial sweeteners.

The exploration of subgroup variability has also been lacking in previous studies, but it has important implications for designing personalized prevention strategies and clinical interventions. In our study, we found that the positive associations between artificial sweeteners and CVD persisted in stratified analyses, which was consistent with the results of the main analyses. Besides, our results indicated that non-whites faced a higher overall CVD risk from artificial sweeteners. Meanwhile, non-obese individuals also tend to be at higher CVD risk from artificial sweeteners, although the interaction P was not significant. Notably, Hannah Gardener et al. also observed that the effect of diet soft drink consumption on vascular events was greater among black participants vs. white (interaction between continuous diet soft drinks/week and black vs. white race HR: 1.06, 95% CI: 1.01,1.11, *p* = 0.03) [9]. Our findings imply that in these particular populations, consumption of artificial sweeteners should be done with greater caution. However, the mechanisms underlying the differences in the impact of artificial sweeteners on CVD outcome risk among different populations are not yet clear. Further verification and mechanistic exploration are needed in the future to understand these differences in greater depth.

Besides, it’s worth noting that the effect size observed is modest. We have made extensive efforts, including a comprehensive adjustment for potential confounders and a series of sensitivity analyses. And the large sample size also provided assurance for the credibility of the results. However, it is still important to use longer follow-up periods, larger sample sizes, more accurate measurement tools, and more sophisticated experimental designs in the future to verify our results and further explore. Meanwhile, although the effect sizes for these associations are modest, they also have important public health implications, given the worldwide prevalence of consumption of artificial sweeteners.

Furthermore, this is the first time we have explored the potential modification role of genetic susceptibility and the mediation role of T2DM in the link between artificial sweeteners and CVD (including overall CVD, CAD, PAD, and HF). Our study showed that the CVD risk associated with artificial sweeteners is independent of genetic susceptibility, and there is no significant additive or multiplicative interaction between genetic susceptibility and artificial sweeteners. But our study on the interaction between artificial sweeteners and genetic susceptibility only included individuals of European ancestry; further exploration in other ethnic populations is needed. We found that 70.0% of the association of artificial sweeteners with overall CVD was mediated by prior T2DM before overall CVD, and 68.2-79.9% of the associations between artificial sweeteners and CAD, PAD, and HF were mediated by prior T2DM before the outcomes. These results revealed that T2DM plays an important mediating role in the association between artificial sweeteners and CVD. Mitigating the intermediate condition (T2DM) contributed to reducing the harmful effect of artificial sweeteners on CVD. These findings also provide mechanistic insight from an epidemiological perspective and corroborate past evidence that links exposure to artificial sweetener intake to incident T2DM [28].

Currently, explanations for the potential mechanisms of the association between artificial sweeteners and CVD are manifold. One leading hypothesis, based on some experimental evidence, suggests that artificial sweeteners may impair glucose homeostasis and induce hyperinsulinemia by altering gut microbiota in both rodent models and humans [53–55]. Artificial sweeteners may also exert effects through intestinal sweet taste receptors, which play a part in insulin secretion and glucose absorption [56]. Some studies have suggested that artificial sweeteners may affect insulin sensitivity and appetite regulation, potentially influencing metabolic health [57–59]. As mentioned above, the significant mediating role of T2DM also seems to support this hypothesis from an epidemiological perspective. However, Joan Serrano et al. reported conflicting results, indicating that short-term saccharin supplementation at maximum acceptable levels does not induce gut microbiota changes or glucose intolerance in healthy humans and mice [60]. A human study suggested that gestational exposure to ASB may contribute to increased infant weight at one-year-old by affecting the structure of gut microbiota structure [61]. Additionally, artificial sweeteners potentially influence the local or systemic inflammatory state by inducing metagenomic alterations in bacterial genes and the subsequent alterations in the production of bacterial byproducts related to anti- or pro-inflammatory response [49, 62, 63]. Recently, extensive in vitro, ex vivo, and preclinical mechanistic studies by Marco Witkowski et al. showed that elevated erythritol levels can directly contribute to heightened platelet reactivity and thrombosis risk by enhancing platelet intracellular calcium release and aggregation in response to multiple agonists [64]. Overall, the mechanisms underlying the association between artificial sweeteners and CVD remain to be further investigated.

### Strengths and limitations

Our study has some strengths. First, this prospective study based on UK Biobank data has a large sample size of over 130,000 participants and detailed information on demographics, lifestyle, medical history, genes, and more. Second, we extensively explored the relationship and subgroup variability between artificial sweetener intake and CVD and CVD mortality. Additionally, we addressed the possibility of confounding through statistical adjustment for a wide range of covariates and a series of sensitivity analyses. Third, we explored for the first time the interaction effect of genetic susceptibility (PRS) on associations between artificial sweetener intake and CVD (including overall CVD, CAD, PAD, and HF), and the further adjustment of genetic influence in the main analysis strengthens the validity of our findings. Fourth, we also explored for the first time the mediating role of T2DM in the association between artificial sweeteners and CVD.

Several limitations should also be acknowledged. First, the observational study cannot demonstrate the causal relationship between artificial sweetener intake and incident CVD and CVD mortality. Second, the interaction effect between artificial sweeteners and genetic susceptibility (PRS) needs to be further explored in different populations outside of cohorts of European ancestry. Third, there is a potential bias in population inclusion and exclusion, such as the exclusion of individuals without 24-hour dietary questionnaire responses, although this is normal in studies based on the UK dietary questionnaire. This exclusion affected the representativeness of our sample and reduced statistical power to some extent. Meanwhile, it should also be noted that UK Biobank participants tend to be healthier than the general population. Fourth, although we conducted sensitivity analyses in populations completing two or more 24-hour dietary questionnaires and obtained consistent results, this methodology still inevitably introduces bias when assessing long-term consumption of artificial sweeteners. It is also important to note that although the 24-hour dietary recall questionnaire is generally reliable and provides an effective means of obtaining relatively specific and detailed dietary information in large population samples, it is essential to acknowledge potential memory biases and subjective estimation errors. Additionally, despite the meticulous design and control of many confounding factors (e.g., demographic characters, socioeconomic characters, diet, lifestyle, and underlying health conditions), other unknown or unmeasured confounders may exist. Fifth, in our study, despite excluding the confounding of ASB - the most significant source of artificial sweetener intake other than tabletop artificial sweeteners, there is still a residual intake in yogurt and other industrial productions that has not been considered. And the intake of artificial sweeteners was assessed only in terms of overall quantity without distinguishing specific types of artificial sweeteners.

## Conclusions

This large-scale perspective study from UK Biobank demonstrated that artificial sweetener intake was significant or marginally significant associated with an increased risk of incident overall CVD, CAD, PAD, and HF. And non-whites were at greater risk of overall CVD incidents from artificial sweeteners, providing important insights into identifying susceptible populations. Our study did not find a significant interaction between genetic susceptibility and artificial sweeteners. The significant mediating role of T2DM on the association between artificial sweeteners and CVD provides mechanistic insight from an epidemiological perspective and suggests that mitigating the intermediate condition (T2DM) contributed to reducing the harmful effect of artificial sweeteners on CVD. Meanwhile, more research on the underlying mechanism is also necessary. Overall, this study expanded our knowledge of the association between artificial sweetener intake and incident CVD in a variety of ways and timely provided important references for future guidelines development and policy formulation.

### Electronic supplementary material

Below is the link to the electronic supplementary material.

Supplementary Material 1

## Data Availability

Data set: Available from the UK Biobank on request (www.ukbiobank.ac.uk).

## Abbreviations

AF: Atrial fibrillation
BMI: Body mass index
CAD: Coronary artery disease
CVD: Cardiovascular disease
CI: Confidence interval
HF: Heart failure
HR: Hazard ratio
LDL-C: Low density lipoprotein cholesterol
MET: Metabolic Equivalent Task
PAD: Peripheral arterial disease
PRS: Polygenic risk score
SBP: Systolic blood pressure
SD: Standard deviation
T2DM: Type 2 diabetes mellitus
