# Supplementary material for: Artificial sweeteners and risk of incident cardiovascular disease and mortality: evidence from UK Biobank
Source: Cardiovasc Diabetol. 2024 Jul 4;23:233. doi: 10.1186/s12933-024-02333-9 (PMC11225337; doi:10.1186/s12933-024-02333-9)

**Artificial Sweeteners and risk of incident cardiovascular disease and mortality: Evidence from UK Biobank**

**Supplementary materials**

**Supplemental Table 1.** Baseline characteristics of the population with and without questionnaire responses

**Supplemental Table 2.** Details of items relevant to the calculation of artificial sweetener intake in dietary questionnaire based on a 24-hour dietary recall

**Supplemental Table 3.** Disease definitions used in the UK Biobank study

**Supplemental Table 4.** Components of the genetic risk score in the study

**Supplemental Table 5.** The percentages of participants with missing covariates

**Supplemental Table 6.** Associations between artificial sweetener intake (coded as a 3-category ordinal variable) and cardiovascular disease mortality, cardiovascular disease, coronary artery disease, peripheral arterial disease, stroke and heart failure, with UK biobank cohort.

**Supplemental Table 7.** Selected pooled baseline characteristics of non-consumers versus higher consumers after propensity score matching

**Supplemental Table 8.** Associations between artificial sweetener intake (non-consumers versus higher consumers) and cardiovascular disease mortality, cardiovascular disease, coronary artery disease, peripheral arterial disease, stroke, heart failure, and atrial fibrillation, after propensity score matching

**Supplemental Table 9.** Stratified analysis of the association between artificial sweetener intake and coronary artery disease incidence

**Supplemental Table 10.** Stratified analysis of the association between artificial sweetener intake and peripheral arterial disease incidence

**Supplemental Table 11.** Stratified analysis of the association between artificial sweetener intake and heart failure incidence

**Supplemental Table 12.** Associations of the risk of incident CAD by polygenic risk score (PRS)

**Supplemental Table 13.** Associations of the risk of incident PAD by polygenic risk score (PRS)

**Supplemental Table 14.** Associations of the risk of incident HF by polygenic risk score (PRS)

**Supplemental Table 15.** Sensitivity analysis 1 on the association of artificial sweetener intake with incident cardiovascular disease mortality, cardiovascular disease, coronary artery disease, peripheral arterial disease, stroke, heart failure, and atrial fibrillation by excluding events that occurred within the first two years of follow-up

**Supplemental Table 16.** Sensitivity analysis 2 on the association of artificial sweetener intake with incident cardiovascular disease mortality, cardiovascular disease, coronary artery disease, peripheral arterial disease, stroke, heart failure, and atrial fibrillation among participants with two or more dietary questionnaires based on a 24-hour dietary recall

**Supplemental Table 17.** Sensitivity analysis 3 on the association of artificial sweetener intake with incident cardiovascular disease mortality, cardiovascular disease, coronary artery disease, peripheral arterial disease, stroke, heart failure, and atrial fibrillation among participants with complete covariate data

**Supplemental Table 18.** Sensitivity analysis 4 on the association of artificial sweetener intake with incident cardiovascular disease mortality, cardiovascular disease, coronary artery disease, peripheral arterial disease, stroke, heart failure, and atrial fibrillation by not excluding participants with baseline diabetes

**Supplemental Table 19.** Sensitivity analysis 5 on the association of artificial sweetener intake with incident cardiovascular disease mortality, cardiovascular disease, coronary artery disease, peripheral arterial disease, stroke, heart failure, and atrial fibrillation by adjusting the consumption clusters

**Supplemental Figure 1.** Restricted cubic spline for the association between artificial sweetener intake and the risk of incident CVD and mortality. Models were adjusted for age, sex, ethnicity, BMI, SBP, LDL-C, Townsend Deprivation Index, cigarette smoking, alcohol consumption, qualification, physical activity, use of lipid-lowering medication, total energy, total sugars, sodium, red and processed meat, fruit, vegetables, saturated fatty acids, monounsaturated fatty acids, and fibre

**Supplemental Table 1.** **Baseline characteristics of the population with and without questionnaire responses**

| **Characteristics** | **Participants without questionnaire responses**  **(n = 291422)** | **Participants with questionnaire responses**  **(n = 210947)** | ***P* value** |
| --- | --- | --- | --- |
|  |  |  |  |
| Age, (years, mean (SD)) | 56.9 (8.2) | 56.1 (7.9) | <0.001 |
| Sex, n (%) |  |  | <0.001 |
| Female | 157099 (53.9) | 116202 (55.1) | - |
| Male | 134323 (46.1) | 94745 (44.9) | - |
| Ethnicity |  |  | <0.001 |
| White | 270941 (93.2) | 201063 (95.4) | - |
| Non-White | 19666 (6.8) | 9799 (4.6) | - |
| BMI (kg/m^2^, mean (SD)) | 27.8 (4.9) | 27.0 (4.7) | <0.001 |
| BMI category (kg/m^2^), n (%) |  |  | <0.001 |
| <25 | 86487 (29.9) | 78491 (37.3) | - |
| 25-30 | 124737 (43.2) | 87325 (41.5) | - |
| ≥ 30 | 77686 (26.9) | 44536 (21.2) | - |
| SBP (mmHg, mean (SD)) | 138.7 (18.9) | 136.7 (18.3) | <0.001 |
| LDL-C (mmol/L, mean (SD)) | 3.6 (0.9) | 3.6 (0.9) | 0.130 |
| Townsend deprivation index (mean (SD)) | -1.1 (3.2) | -1.6 (2.9) | <0.001 |
| MET (minutes/week, mean (SD)) | 1093.7 (1136.7) | 971.9 (1015.5) | <0.001 |
| MET category (minutes/week), n (%) |  |  | <0.001 |
| <600 | 105441 (47.2) | 89312 (50.0) | - |
| 600-1500 | 72280 (32.3) | 59929 (33.5) | - |
| ≥ 1500 | 45814 (20.5) | 29497 (16.5) | - |
| Cigarette smoking, n (%) |  |  | <0.001 |
| Never | 154431 (53.4) | 118970 (56.6) | - |
| Previous | 98155 (34.0) | 74854 (35.6) | - |
| Current | 36415 (12.6) | 16546 (7.9) | - |
| Alcohol consumption, n (%) |  |  | <0.001 |
| Not current | 27265 (9.4) | 13359 (6.3) | - |
| Two or fewer times a week | 146418 (50.5) | 96666 (45.9) | - |
| Three or more times a week | 116414 (40.1) | 100745 (47.8) | - |
| Education level, n (%) |  |  | <0.001 |
| No qualification | 67178 (23.8) | 18079 (8.6) | - |
| Any other qualification | 143776 (50.9) | 102100 (48.6) | - |
| Degree or above | 71380 (25.3) | 89724 (42.7) | - |
| Use of lipid-lowering medication |  |  | <0.001 |
| No | 229308 (80.8) | 177581 (84.6) | - |
| Yes | 54466 (19.2) | 32409 (15.4) | - |

Townsend deprivation index=a composite area-level measure of deprivation based on unemployment, non-car ownership, non-home ownership, and household overcrowding; a higher score indicates higher deprivation.

BMI, body mass index; LDL-C, low density lipoprotein cholesterol; MET, Metabolic Equivalent Task; SBP, systolic blood pressure; SD, standard deviation.

**Supplemental Table 2. Details of items relevant to the calculation of artificial sweetener intake in dietary questionnaire based on a 24-hour dietary recall**

| **Questions Field** | **Note** | **Field ID** | **Code** |
| --- | --- | --- | --- |
| **All items of artificial sweetener** | | | |
| Intake of artificial sweetener added to coffee | How many teaspoons/tablets of sweetener (e.g. Canderel) did you add to your coffee (per drink)? | 100380 | 100002 |
| Intake of artificial sweetener added to tea | How many teaspoons/tablets of sweetener (e.g. Canderel) did you add to your tea/infusion (per drink)? | 100500 |  |
| Intake of artificial sweetener added to cereal | How much sweetener (e.g. Canderel) did you add to your cereal or porridge (per bowl)? | 100910 |  |
| **Items of coffee consumption** | | | |
| Instant coffee intake | How many cups/mugs of instant coffee did you drink yesterday? | 100250 | 100006 |
| Filtered coffee intake | How many cups/mugs of filter/americano/cafetiere coffee did you drink yesterday? | 100270 |  |
| Cappuccino intake | How many cups/mugs of cappuccino did you drink yesterday? | 100290 |  |
| Latte intake | How many cups/mugs of latte did you drink yesterday? | 100300 |  |
| Espresso intake | How many cups/mugs of espresso did you drink yesterday? | 100310 |  |
| Other coffee type | How many cups/mugs of other coffee drinks did you drink yesterday? | 100330 |  |
| **Items of tea consumption** | | | |
| Standard tea intake | How many cups/mugs of standard tea (e.g. Tetley, PG Tips, Assam, Darjeeling) did you drink yesterday? | 100400 | 100007 |
| Rooibos tea intake | How many cups/mugs of rooibos/redbush tea (e.g. Tick Tock) did you drink yesterday? | 100410 |  |
| Green tea intake | How many cups/mugs of green tea did you drink yesterday? | 100420 |  |
| Herbal tea intake | How many cups/mugs of herbal or fruit tea (infusion) did you drink yesterday? | 100430 |  |
| Other tea intake | How many cups/mugs of other tea or infusion did you drink yesterday? | 100440 |  |
| **Items of cereal** **consumption** | | | |
| Porridge intake | How many bowls of porridge, hot oat cereal (e.g. Ready Brek)? | 100770 | 100001 |
| Muesli intake | How many bowls of muesli? | 100800 |  |
| Oat crunch intake | How many bowls of sweetened oat crunch type cereal (e.g. Jordans Country Crisp, chocolate nut clusters)? | 100810 |  |
| Sweetened cereal intake | How many bowls of other sweetened cereals (e.g. Ricicles, Honey Nut Cornflakes, Coco Pops)? | 100820 |  |
| Plain cereal intake | How many bowls of plain cereals (e.g. Cornflakes, Rice Krispies, Special K)? | 100830 |  |
| Bran cereal intake | How many bowls of bran cereals (e.g. bran flakes, All Bran)? | 100840 |  |
| Whole-wheat cereal intake | How many bowls of whole-wheat cereals (e.g. Weetabix, Shredded Wheat, Shreddies)? | 100850 |  |
| Other cereal intake | How many bowls of other cereal? | 100860 |  |

**Supplemental Table 3. Disease definitions (ICD) used in the UK Biobank study**

|  | **ICD-9 (Field ID: 41271)** | **ICD-10 (Field ID: 41270)** |
| --- | --- | --- |
| CVD mortality |  | I00-I99 |
| CVD | 390-459 | I00-I99 |
| CAD | 410, 411, 412 | I21, I22, I23, I24.1, I25.2 |
| PAD | 4400, 4402, 4438, 4439 | I70, I70.0, I70.00, I70.01, I70.2, I70.20, I70.21, I70.8, I70.80, I70.81, I70.9, I70.90, I70.91, I73.8, I73.9 |
| Stroke | 430, 431, 434, 436 | I60, I61, I63, I64 |
| HF | 428, 4280, 4281, 4289 | I50 |
| AF | 4273 | I48 |
| Diabetes | 250 | E10, E11, E12, E13, E14 |
| T2DM | 25000, 25010, 25020, 25090 | E11 |

Variable definitions constructed using ICD-9 and ICD-10 fields with choice-, disease- or procedure-specific codes between brackets are shown.

AF, atrial fibrillation; CVD, cardiovascular disease; CAD, coronary artery disease; PAD, peripheral arterial disease; HF, heart failure; ICD, international classification of diseases; T2DM, type 2 diabetes mellitus.

**Supplemental Table 4. Components of the genetic risk score in the study**

| **SNP** | **CHR** | **BP** | **Effect allele** | **Non-effect allele** | **Beta** | **EAF** | **P** |
| --- | --- | --- | --- | --- | --- | --- | --- |
| **CAD** | | | | | | | |
| rs11591147 | 1 | 55505647 | G | T | 0.2209 | 0.98355 | 2.84E-10 |
| rs56170783 | 1 | 57016131 | A | C | 0.10435 | 0.91462 | 2.14E-12 |
| rs7528419 | 1 | 109817192 | A | G | 0.1086 | 0.78215 | 3.77E-27 |
| rs11810571 | 1 | 151762308 | G | C | 0.05836 | 0.80704 | 2.21E-08 |
| rs6689306 | 1 | 154395946 | A | G | 0.05023 | 0.43525 | 1.46E-09 |
| rs67180937 | 1 | 222823743 | G | T | 0.07106 | 0.68434 | 8.45E-14 |
| rs16986953 | 2 | 19942473 | A | G | 0.10531 | 0.0731 | 4.77E-10 |
| rs585967 | 2 | 21270554 | C | A | 0.06576 | 0.84373 | 2.76E-08 |
| rs4299376 | 2 | 44072576 | G | T | 0.05546 | 0.31916 | 5.65E-10 |
| rs7568458 | 2 | 85788175 | A | T | 0.0609 | 0.45164 | 2.39E-13 |
| rs17678683 | 2 | 145286559 | G | T | 0.07653 | 0.08904 | 1.15E-07 |
| rs114123510 | 2 | 203831212 | A | T | 0.11811 | 0.11588 | 2.88E-19 |
| rs1250229 | 2 | 216304384 | T | C | 0.0694 | 0.26201 | 1.85E-13 |
| rs13003675 | 2 | 233584109 | T | C | 0.04194 | 0.3609 | 1.72E-06 |
| rs7623687 | 3 | 49448566 | A | C | 0.07155 | 0.85867 | 3.72E-09 |
| rs62265630 | 3 | 124475201 | G | T | 0.06717 | 0.14068 | 1.00E-08 |
| rs12493885 | 3 | 153839866 | C | G | 0.07094 | 0.86297 | 3.29E-08 |
| rs72627509 | 4 | 57839051 | G | C | 0.0537 | 0.19944 | 8.10E-08 |
| rs10857147 | 4 | 81181072 | T | A | 0.05404 | 0.2772 | 8.96E-09 |
| rs7678555 | 4 | 120909501 | C | A | 0.0483 | 0.28396 | 1.43E-07 |
| rs6841581 | 4 | 148401190 | A | G | 0.06834 | 0.15321 | 4.57E-10 |
| rs2306556 | 4 | 156638573 | A | G | 0.06329 | 0.81589 | 1.22E-09 |
| rs77335401 | 5 | 131759825 | C | T | 0.04908 | 0.11608 | 7.62E-05 |
| rs742115 | 6 | 11327021 | C | T | 0.03585 | 0.48448 | 2.86E-05 |
| rs9349379 | 6 | 12903957 | G | A | 0.10544 | 0.40681 | 9.95E-36 |
| rs6909752 | 6 | 22612629 | A | G | 0.05002 | 0.34808 | 5.59E-09 |
| rs3130683 | 6 | 31888367 | T | C | 0.07703 | 0.85979 | 2.77E-08 |
| rs4472337 | 6 | 34769765 | T | C | 0.05533 | 0.15511 | 2.42E-06 |
| rs56015508 | 6 | 39152041 | C | A | 0.05385 | 0.79374 | 1.08E-07 |
| rs12202017 | 6 | 134173151 | A | G | 0.06636 | 0.70173 | 6.02E-14 |
| rs10455872 | 6 | 161010118 | G | A | 0.27149 | 0.06498 | 1.71E-49 |
| rs2107595 | 7 | 19049388 | A | G | 0.07422 | 0.18163 | 3.41E-13 |
| rs112370447 | 7 | 107176780 | T | C | 0.04547 | 0.28035 | 9.62E-07 |
| rs11556924 | 7 | 129663496 | C | T | 0.067 | 0.65726 | 6.26E-13 |
| rs3918226 | 7 | 150690176 | T | C | 0.12529 | 0.07106 | 1.58E-12 |
| rs2083636 | 8 | 19865263 | T | G | 0.05136 | 0.74225 | 6.44E-08 |
| rs2954029 | 8 | 126490972 | A | T | 0.06039 | 0.54147 | 5.24E-13 |
| rs2891168 | 9 | 22098619 | G | A | 0.17327 | 0.48703 | 1.28E-101 |
| rs111245230 | 9 | 113169775 | C | T | 0.10937 | 0.0357 | 8.29E-07 |
| rs507666 | 9 | 136149399 | A | G | 0.07404 | 0.19249 | 1.34E-12 |
| rs1887318 | 10 | 30321598 | T | C | 0.05771 | 0.42773 | 4.12E-12 |
| rs1870634 | 10 | 44480811 | G | T | 0.06182 | 0.64791 | 5.51E-13 |
| rs2246942 | 10 | 91004886 | G | A | 0.07626 | 0.34861 | 3.51E-16 |
| rs11191416 | 10 | 104604916 | T | G | 0.07287 | 0.88991 | 5.58E-09 |
| rs10840293 | 11 | 9751196 | A | G | 0.04868 | 0.55182 | 6.88E-09 |
| rs3993105 | 11 | 13303071 | T | C | 0.04707 | 0.68628 | 1.06E-07 |
| rs2839812 | 11 | 103673294 | T | A | 0.05954 | 0.30735 | 1.99E-11 |
| rs964184 | 11 | 116648917 | G | C | 0.05083 | 0.16343 | 4.68E-06 |
| rs2229357 | 12 | 57843711 | G | A | 0.04684 | 0.76448 | 3.39E-06 |
| rs2681472 | 12 | 90008959 | G | A | 0.06597 | 0.18906 | 7.63E-11 |
| rs10774625 | 12 | 111910219 | A | G | 0.06422 | 0.4906 | 9.22E-14 |
| rs11830157 | 12 | 118265441 | G | T | 0.02682 | 0.38209 | 0.001652 |
| rs2244608 | 12 | 121416988 | G | A | 0.05127 | 0.33509 | 2.32E-09 |
| rs11057830 | 12 | 125307053 | A | G | 0.06864 | 0.14653 | 4.24E-09 |
| rs1924981 | 13 | 29022645 | T | C | 0.04567 | 0.33395 | 0.000000186 |
| rs11617955 | 13 | 110818102 | T | A | 0.08738 | 0.8911 | 4.14E-10 |
| rs10139550 | 14 | 100145710 | G | C | 0.05109 | 0.4214 | 1.84E-09 |
| rs72743461 | 15 | 67441750 | C | A | 0.07124 | 0.78308 | 4.81E-12 |
| rs7164479 | 15 | 79123054 | T | C | 0.07211 | 0.57817 | 6.38E-18 |
| rs2083460 | 15 | 89574484 | T | C | 0.07164 | 0.88539 | 0.000000141 |
| rs2071382 | 15 | 91428197 | T | C | 0.0617 | 0.46364 | 7.14E-13 |
| rs247616 | 16 | 56989590 | C | T | 0.0438 | 0.67831 | 0.00000101 |
| rs7500448 | 16 | 83045790 | A | G | 0.05905 | 0.76263 | 5.14E-09 |
| rs9897596 | 17 | 17593453 | T | C | 0.03949 | 0.52319 | 0.00000313 |
| rs4643373 | 17 | 47123423 | T | C | 0.04584 | 0.72385 | 0.0000012 |
| rs8068952 | 17 | 59286644 | G | C | 0.06961 | 0.2282 | 1.41E-09 |
| rs116843064 | 19 | 8429323 | G | A | 0.15908 | 0.98016 | 0.000000287 |
| rs6511720 | 19 | 11202306 | G | T | 0.12826 | 0.88413 | 7.88E-22 |
| rs10417115 | 19 | 33386556 | C | T | 0.0684 | 0.06087 | 0.0000225 |
| rs8108632 | 19 | 41854534 | T | A | 0.0478 | 0.45251 | 5.88E-08 |
| rs7412 | 19 | 45412079 | C | T | 0.1432 | 0.92156 | 2.17E-19 |
| rs1964272 | 19 | 46190268 | G | A | 0.04413 | 0.51766 | 0.000000229 |
| rs28451064 | 21 | 35593827 | A | G | 0.13279 | 0.12444 | 2.62E-23 |
| rs180803 | 22 | 24658858 | G | T | 0.16536 | 0.97722 | 7.09E-10 |
| **PAD** | | | | | | | |
| rs7528419 | 1 | 109817192 | A | G | 0.029383778 | 0.7721 | 3E-11 |
| rs6025 | 1 | 169519049 | T | C | 0.079181246 | 0.0265 | 2E-12 |
| rs3130968 | 6 | 31065071 | T | C | 0.029383778 | 0.1371 | 3E-10 |
| rs118039278 | 6 | 160985526 | A | G | 0.100370545 | 0.0643 | 2E-43 |
| rs2107595 | 7 | 19049388 | A | G | 0.033423755 | 0.187 | 2E-11 |
| rs4722172 | 7 | 22786532 | G | A | 0.033423755 | 0.1957 | 4E-11 |
| rs322 | 8 | 19819217 | A | C | 0.025305865 | 0.702 | 0.000000003 |
| rs1537372 | 9 | 22103183 | T | G | 0.049218023 | 0.4176 | 4E-39 |
| rs505922 | 9 | 136149229 | C | T | 0.025305865 | 0.3371 | 7E-11 |
| rs7903146 | 10 | 114758349 | T | C | 0.025305865 | 0.2937 | 4E-11 |
| rs7476 | 11 | 46342834 | C | A | 0.025305865 | 0.3763 | 8E-10 |
| rs566125 | 11 | 102710471 | T | C | 0.033423755 | 0.1269 | 0.000000004 |
| rs4842266 | 12 | 79951566 | G | A | 0.025305865 | 0.4077 | 0.000000001 |
| rs11066301 | 12 | 112871372 | G | A | 0.025305865 | 0.4109 | 3E-11 |
| rs1975514 | 13 | 110828891 | C | T | 0.021189299 | 0.3534 | 8E-10 |
| rs55784307 | 14 | 70501364 | A | C | 0.025305865 | 0.1817 | 0.00000003 |
| rs10851907 | 15 | 78915864 | A | G | 0.025305865 | 0.4105 | 1E-13 |
| rs62084752 | 17 | 66089393 | C | G | 0.029383778 | 0.2171 | 2E-10 |
| rs138294113 | 19 | 11191729 | C | T | 0.037426498 | 0.879 | 1E-10 |
| **HF** | | | | | | | |
| rs660240 | 1 | 109817838 | T | C | -0.0611 | 0.2128 | 3.25E-10 |
| rs17042102 | 4 | 111668626 | A | G | 0.1103 | 0.115 | 5.71E-20 |
| rs11745324 | 5 | 137012171 | A | G | -0.0528 | 0.2277 | 2.35E-08 |
| rs55730499 | 6 | 161005610 | T | C | 0.1058 | 0.0694 | 1.83E-11 |
| rs4135240 | 6 | 36647680 | T | C | 0.0486 | 0.6589 | 6.84E-09 |
| rs140570886 | 6 | 161013013 | T | C | -0.2136 | 0.9842 | 7.69E-11 |
| rs1556516 | 9 | 22100176 | C | G | 0.0622 | 0.4845 | 1.57E-15 |
| rs600038 | 9 | 136151806 | T | C | -0.0569 | 0.7909 | 3.68E-09 |
| rs17617337 | 10 | 121426884 | T | C | -0.0561 | 0.2208 | 3.65E-09 |
| rs4746140 | 10 | 75417249 | C | G | -0.0666 | 0.154 | 1.1E-09 |
| rs4766578 | 12 | 111904371 | A | T | -0.0433 | 0.5287 | 0.000000049 |
| rs56094641 | 16 | 53806453 | A | G | -0.0454 | 0.5842 | 1.21E-08 |

SNP, single nucleotide polymorphism; EAF, effect allele frequency.

**Supplementary Table 5. The percentages of participants with missing covariates**

| **Covariates** | **Percentage of missing data** | |
| --- | --- | --- |
| Sex | 0.00% | |
| Age | 0.00% | |
| Ethnicity | 0.05% | |
| BMI | 0.23% | |
| SBP | 3.55% | |
| LDL-C | 5.67% | |
| Cigarette smoking | 0.24% | |
| Alcohol consumption | 0.08% | |
| Education level | 0.46% | |
| Townsend deprivation index | 0.11% | |
| Physical activity | 14.99% | |
| Lipid-lowering medication | 0.45% | |
| Total energy | 0.00% | |
| Total sugars | 0.00% | |
| Sodium | 0.00% | |
| Red and processed meat | 0.58% | |
| Fruit | 12.10% | |
| Vegetables | 6.98% | |
| Saturated fatty acids | 0.00% | |
| Monounsaturated fatty acids | 0.00% | |
| Fibre | 0.00% | |
| Added sugars and preserves | 0.00% | |
|  | |  |
| Overall | 2.07% |  |

BMI, body mass index; LDL-C, low density lipoprotein cholesterol; SBP, systolic blood pressure.

**Supplemental Table 6. Associations between artificial sweetener intake (coded as a 3-category ordinal variable) and cardiovascular disease mortality, cardiovascular disease, coronary artery disease, peripheral arterial disease, stroke and heart failure, with UK biobank cohort.**

|  |  | **Non-consumers** | **Lower consumers (≤ 4 teaspoons(tablets)/day)** | **Higher consumers (> 4 teaspoons(tablets)/day)** |
| --- | --- | --- | --- | --- |
|  |  |  |  |  |
|  | Total No. of Participants | 120858 | 6993 | 5434 |
| CVD mortality | Cases/Person-years | 861/1321701 | 68/76469 | 62/59278 |
|  | HR (95% CI); *P* | ref | 1.172 (0.914,1.503);  0.210 | 1.095 (0.844,1.422);  0.494 |
|  |  |  |  |  |
| CVD | Cases/Person-years | 33822/1139546 | 2355/63463 | 2083/47874 |
|  | HR (95% CI); *P* | ref | 1.063 (1.019,1.108);  0.005 | 1.106 (1.058,1.157);  <0.001 |
|  |  |  |  |  |
| CAD | Cases/Person-years | 2347/1310829 | 134/75860 | 174/58511 |
|  | HR (95% CI); *P* | ref | 0.864 (0.726,1.030);  0.102 | 1.163 (0.995,1.360);  0.058 |
|  |  |  |  |  |
| PAD | Cases/Person-years | 718/1319256 | 66/76271 | 73/59043 |
|  | HR (95% CI); *P* | ref | 1.293 (1.004,1.667);  0.047 | 1.377 (1.078,1.759);  0.010 |
|  |  |  |  |  |
| Stroke | Cases/Person-years | 1714/1315523 | 100/76065 | 98/58868 |
|  | HR (95% CI); *P* | ref | 0.901 (0.735,1.103);  0.312 | 0.995 (0.810,1.223);  0.965 |
|  |  |  |  |  |
| HF | Cases/Person-years | 1686/1316375 | 130/76027 | 133/58872 |
|  | HR (95% CI); *P* | ref | 1.105 (0.923,1.322);  0.277 | 1.169 (0.977,1.398);  0.087 |
|  |  |  |  |  |
| AF | Cases/Person-years | 5249/1299857 | 345/75086 | 295/57971 |
|  | HR (95% CI); *P* | ref | 1.001 (0.897,1.117);  0.990 | 0.939 (0.834,1.057);  0.294 |
|  |  |  |  |  |

Data are hazard ratios (95% CIs). Models were adjusted for age, sex, ethnicity, BMI, SBP, LDL-C, Townsend Deprivation Index, cigarette smoking, alcohol consumption, qualification, physical activity, use of lipid-lowering medication, total energy, total sugars, sodium, red and processed meat, fruit, vegetables, saturated fatty acids, monounsaturated fatty acids, and fibre. *P* < 0.05 was considered statistically significant.

AF, atrial fibrillation; BMI, body mass index; CAD, coronary artery disease; CVD, cardiovascular disease; CI, confidence interval; HF, heart failure; HR, hazard ratio; LDL-C, low density lipoprotein cholesterol; PAD, peripheral arterial disease; SBP, systolic blood pressure.

**Supplementary Table 7.** **Selected pooled baseline characteristics of non-consumers versus lower consumers and non-consumers versus higher consumers after propensity score matching**

| **Characteristics** | **Non-consumers**  **(n = 16257)** | **Higher Consumers (n = 5419)** | **Standardized difference** |
| --- | --- | --- | --- |
| Age, (years, mean (SD)) | 57.4 (7.6) | 57.4 (7.6) | 0.002 |
| Sex, n (%) |  |  | 0.003 |
| Female | 8555 (52.6) | 2860 (52.8) | - |
| Male | 7702 (47.4) | 2559 (47.2) | - |
| Ethnicity |  |  | 0.011 |
| Non-White | 619 (3.8) | 195 (3.6) | - |
| White | 15634 (96.2) | 5224 (96.4) | - |
| BMI (kg/m^2^, mean (SD)) | 27.7 (4.4) | 27.9 (4.6) | 0.038 |
| BMI category (kg/m^2^), n (%) |  |  | 0.020 |
| <25 | 4213 (25.9) | 1448 (26.7) | - |
| 25-30 | 7743 (47.6) | 2535 (46.8) | - |
| ≥ 30 | 4301 (26.5) | 1436 (26.5) | - |
| SBP (mmHg, mean (SD)) | 138.4 (18.2) | 138.2 (18.1) | 0.010 |
| LDL-C (mmol/L, mean (SD)) | 3.6 (0.8) | 3.6 (0.8) | 0.010 |
| Townsend deprivation index (mean (SD)) | -1.4 (3.0) | -1.5 (2.9) | 0.009 |
| MET (minutes/week, mean (SD)) | 1031.8 (1077.8) | 1007.0 (1063.0) | 0.023 |
| MET category (minutes/week), n(%) |  |  | 0.038 |
| <600 | 6610 (40.7) | 2242 (41.4) | - |
| 600-1500 | 7069 (43.5) | 2263 (41.8) | - |
| ≥ 1500 | 2578 (15.9) | 914 (16.9) | - |
| Cigarette smoking, n (%) |  |  | 0.008 |
| Never | 6930 (42.7) | 2307 (42.7) | - |
| Previous | 7072 (43.6) | 2343 (43.3) | - |
| Current | 2227 (13.7) | 757 (14.0) | - |
| Alcohol consumption, n (%) |  |  | 0.005 |
| Not current | 1217 (7.5) | 413 (7.6) | - |
| Two or fewer times a week | 8496 (52.3) | 2834 (52.3) | - |
| Three or more times a week | 6531 (40.2) | 2171 (40.1) | - |
| Education level, n (%) |  |  | 0.007 |
| No qualification | 2236 (13.8) | 745 (13.8) | - |
| Any other qualification | 9394 (58.2) | 3123 (57.9) | - |
| Degree or above | 4521 (28.0) | 1527 (28.3) | - |
| Use of lipid-lowering medication, n(%) |  |  | 0.010 |
| No | 13709 (84.7) | 4548 (84.4) | - |
| Yes | 2469 (15.3) | 842 (15.6) | - |
| Total energy (kj, mean (SD)) | 8632.7 (2651.8) | 8615.9 (2584.5) | 0.006 |
| Total sugars (mean (SD)) | 125.3 (51.5) | 125.1 (51.1) | 0.004 |
| Sodium(mg, mean (SD)) | 2,019.8 (880.5) | 2,022.9 (818.9) | 0.004 |
| Red and processed meat (mean (SD)) | 13.0 (2.7) | 13.0 (2.6) | 0.015 |
| Fruit (pieces/day, mean (SD)) | 2.3 (1.5) | 2.3 (1.6) | <0.001 |
| Vegetable (tablespoons/day, mean (SD)) | 4.9 (3.1) | 4.9 (3.3) | 0.001 |
| Saturated fatty acids (g, mean (SD)) | 27.0 (12.6) | 26.9 (12.4) | 0.006 |
| Monounsaturated fatty acids (g, mean (SD)) | 26.3 (11.6) | 26.3 (11.4) | 0.003 |
| Fibre (g, mean (SD)) | 17.2 (6.9) | 17.3 (6.7) | 0.007 |

Townsend deprivation index=a composite area-level measure of deprivation based on unemployment, non-car ownership, non-home ownership, and household overcrowding; a higher score indicates higher deprivation.

BMI, body mass index; LDL-C, low density lipoprotein cholesterol; MET, Metabolic Equivalent Task; SBP, systolic blood pressure; SD, standard deviation.

**Supplementary Table 8.** **Associations between artificial sweetener intake (non-consumers versus lower consumers and non-consumers versus higher consumers) and cardiovascular disease mortality, cardiovascular disease, coronary artery disease, peripheral arterial disease, stroke, heart failure, and atrial fibrillation, after propensity score matching**

|  |  | **Non-consumer** | **Higher Consumers** |
| --- | --- | --- | --- |
|  |  | **(n = 16257)** | **(n = 5419)** |
|  | Total No. of Participants | 16257 | 5419 |
| CVD mortality | Cases/Person-years | 165/178165 | 61/59127 |
|  | HR (95% CI); *P* | ref | 1.113 (0.829,1.493);  0.477 |
| Overall CVD | Cases/Person-years | 5830/146126 | 2074/47789 |
|  | HR (95% CI); *P* | ref | 1.103 (1.049,1.159);  <0.001 |
| CAD | Cases/Person-years | 418/176194 | 172/58379 |
|  | HR (95% CI); *P* | ref | 1.251 (1.047,1.494);  0.014 |
| PAD | Cases/Person-years | 139/177684 | 71/58898 |
|  | HR (95% CI); *P* | ref | 1.560 (1.171,2.077);  0.002 |
| Stroke | Cases/Person-years | 302/177063 | 98/58718 |
|  | HR (95% CI); *P* | ref | 0.992 (0.790,1.246);  0.946 |
| HF | Cases/Person-years | 310/177250 | 131/58725 |
|  | HR (95% CI); *P* | ref | 1.286 (1.048,1.578);  0.016 |
| AF | Cases/Person-years | 923/174171 | 292/57837 |
|  | HR (95% CI); *P* | ref | 0.949 (0.832,1.083);  0.436 |

Data are hazard ratios (95% CIs). Models were adjusted for age, sex, ethnicity, BMI, SBP, LDL-C, Townsend Deprivation Index, cigarette smoking, alcohol consumption, qualification, physical activity, use of lipid-lowering medication, total energy, total sugars, sodium, red and processed meat, fruit, vegetables, saturated fatty acids, monounsaturated fatty acids, and fibre. *P* < 0.05 was considered statistically significant.

AF, atrial fibrillation; BMI, body mass index; CAD, coronary artery disease; CVD, cardiovascular disease; CI, confidence interval; HF, heart failure; HR, hazard ratio; LDL-C, low density lipoprotein cholesterol; PAD, peripheral arterial disease; SBP, systolic blood pressure

**Supplemental Table 9. Stratified analysis of the association between artificial sweetener intake and coronary artery disease incidence**

| **Subgroup** | **Total No. of Participants** | **Cases/Person-Years** | **HR (95% CI)** | ***P* for interaction** |
| --- | --- | --- | --- | --- |
|  |  |  |  |  |
| **Age at baseline, years** | | | | |
| ≥65 | 19575 | 744/206826 | 0.994 (0.962,1.028) | 0.058 |
| <65 | 113710 | 1911/1238374 | 1.028 (1.008,1.048) |  |
| **Sex** | | | | |
| Men | 58225 | 1877/625339 | 1.014 (0.994,1.034) | 0.312 |
| Women | 75060 | 778/819862 | 1.030 (0.998,1.063) |  |
| **Ethnicity** | | | | |
| White | 127080 | 2552/1375326 | 1.017 (0.999,1.034) | 0.572 |
| Others | 6205 | 103/69874 | 1.046 (0.960,1.139) |  |
| **Obesity, kg/m^2^** | | | | |
| BMI ≥30 | 21123 | 567/230134 | 1.033 (1.006,1.061) | 0.281 |
| BMI <30 | 112162 | 2088/1215066 | 1.010 (0.989,1.032) |  |
| **Smoking status** | | | | |
| Never | 78130 | 1268/851735 | 0.999 (0.965,1.034) | 0.076 |
| Previous | 44624 | 1019/480373 | 1.030 (1.008,1.054) |  |
| Current | 10531 | 368/113093 | 1.008 (0.972,1.045) |  |
| **Alcohol consumption** | | | | |
| Not current | 7570 | 178/82602 | 1.022 (0.967,1.081) | 0.897 |
| Two or fewer times a week | 59478 | 1203/648172 | 1.021 (0.998,1.044) |  |
| Three or more times a week | 66237 | 1274/714427 | 1.015 (0.987,1.044) |  |
| **Qualification** | | | | |
| No qualification | 9794 | 346/106847 | 0.998 (0.960,1.037) | 0.711 |
| Any other qualification | 62885 | 1331/686417 | 1.022 (1.000,1.045) |  |
| Degree or above | 60606 | 978/651937 | 1.022 (0.988,1.058) |  |
| **Physical activity, minutes/week** | | | | |
| MET: <600 | 56414 | 1124/609154 | 1.024 (0.998,1.050) | 0.870 |
| MET: 600-1500 | 57505 | 1101/624470 | 1.009 (0.981,1.037) |  |
| MET: ≥1500 | 19366 | 430/211577 | 1.021 (0.985,1.058) |  |

Data are hazard ratios (95% CIs). Models were adjusted for age, sex, ethnicity, BMI, SBP, LDL-C, Townsend Deprivation Index, cigarette smoking, alcohol consumption, qualification, physical activity, use of lipid-lowering medication, total energy, total sugars, sodium, red and processed meat, fruit, vegetables, saturated fatty acids, monounsaturated fatty acids, and fibre.

BMI, body mass index; LDL-C, low density lipoprotein cholesterol; CI, confidence interval; HR, hazard ratio; MET, metabolic equivalent task; SBP, systolic blood pressure.

**Supplemental Table 10. Stratified analysis of the association between artificial sweetener intake and peripheral arterial disease incidence**

| **Subgroup** | **Total No. of Participants** | **Cases/Person-Years** | **HR (95% CI)** | ***P* for interaction** |
| --- | --- | --- | --- | --- |
|  |  |  |  |  |
| **Age at baseline, years** | | | | |
| ≥65 | 19575 | 306/209114 | 1.045 (1.005,1.086) | 0.723 |
| <65 | 113710 | 551/1245456 | 1.028 (0.995,1.062) |  |
| **Sex** | | | | |
| Men | 58225 | 513/632628 | 1.036 (1.005,1.068) | 0.791 |
| Women | 75060 | 344/821942 | 1.030 (0.987,1.076) |  |
| **Ethnicity** | | | | |
| White | 127080 | 827/1384273 | 1.030 (1.003,1.056) | 0.060 |
| Others | 6205 | 30/70296 | 1.166 (1.053,1.291) |  |
| **Obesity, kg/m^2^** | | | | |
| BMI ≥30 | 21123 | 197/232108 | 1.018 (0.972,1.065) | 0.310 |
| BMI <30 | 112162 | 660/1222461 | 1.043 (1.013,1.075) |  |
| **Smoking status** | | | | |
| Never | 78130 | 289/856451 | 1.062 (1.009,1.117) | 0.171 |
| Previous | 44624 | 364/483896 | 1.046 (1.010,1.084) |  |
| Current | 10531 | 204/114222 | 1.000 (0.951,1.051) |  |
| **Alcohol consumption** | | | | |
| Not current | 7570 | 60/83125 | 0.986 (0.892,1.090) | 0.496 |
| Two or fewer times a week | 59478 | 351/652492 | 1.040 (1.005,1.077) |  |
| Three or more times a week | 66237 | 446/718953 | 1.039 (0.999,1.079) |  |
| **Qualification** | | | | |
| No qualification | 9794 | 144/107996 | 0.963 (0.898,1.031) | 0.012 |
| Any other qualification | 62885 | 438/691062 | 1.065 (1.034,1.096) |  |
| Degree or above | 60606 | 275/655512 | 1.016 (0.955,1.080) |  |
| **Physical activity, minutes/week** | | | | |
| MET: <600 | 56414 | 350/613216 | 1.055 (1.017,1.095) | 0.059 |
| MET: 600-1500 | 57505 | 370/628234 | 1.042 (1.004,1.082) |  |
| MET: ≥1500 | 19366 | 137/213120 | 0.957 (0.881,1.040) |  |

Data are hazard ratios (95% CIs). Models were adjusted for age, sex, ethnicity, BMI, SBP, LDL-C, Townsend Deprivation Index, cigarette smoking, alcohol consumption, qualification, physical activity, use of lipid-lowering medication, total energy, total sugars, sodium, red and processed meat, fruit, vegetables, saturated fatty acids, monounsaturated fatty acids, and fibre.

BMI, body mass index; LDL-C, low density lipoprotein cholesterol; CI, confidence interval; HR, hazard ratio; MET, metabolic equivalent task; SBP, systolic blood pressure.

**Supplemental Table 11. Stratified analysis of the association between artificial sweetener intake and heart failure incidence**

| **Subgroup** | **Total No. of Participants** | **Cases/Person-Years** | **HR (95% CI)** | ***P* for interaction** |
| --- | --- | --- | --- | --- |
|  |  |  |  |  |
| **Age at baseline, years** | | | | |
| ≥65 | 19575 | 764/207778 | 1.019 (0.990,1.049) | 0.679 |
| <65 | 113710 | 1185/1243496 | 1.019 (0.994,1.044) |  |
| **Sex** | | | | |
| Men | 58225 | 1188/630352 | 1.025 (1.003,1.049) | 0.583 |
| Women | 75060 | 761/820922 | 1.003 (0.970,1.038) |  |
| **Ethnicity** | | | | |
| White | 127080 | 1882/1381080 | 1.017 (0.998,1.037) | 0.442 |
| Others | 6205 | 67/70194 | 1.051 (0.955,1.156) |  |
| **Obesity, kg/m^2^** | | | | |
| BMI ≥30 | 21123 | 539/231066 | 1.029 (1.001,1.058) | 0.583 |
| BMI <30 | 112162 | 1410/1220208 | 1.011 (0.985,1.037) |  |
| **Smoking status** | | | | |
| Never | 78130 | 909/854400 | 1.022 (0.988,1.057) | 0.231 |
| Previous | 44624 | 795/482722 | 1.029 (1.002,1.055) |  |
| Current | 10531 | 245/114153 | 0.986 (0.939,1.036) |  |
| **Alcohol consumption** | | | | |
| Not current | 7570 | 148/82893 | 0.995 (0.928,1.068) | 0.277 |
| Two or fewer times a week | 59478 | 835/651031 | 1.032 (1.007,1.058) |  |
| Three or more times a week | 66237 | 966/717350 | 1.009 (0.977,1.041) |  |
| **Qualification** | | | | |
| No qualification | 9794 | 289/107560 | 1.019 (0.982,1.058) | 0.588 |
| Any other qualification | 62885 | 965/689355 | 1.009 (0.982,1.037) |  |
| Degree or above | 60606 | 695/654360 | 1.031 (0.994,1.070) |  |
| **Physical activity, minutes/week** | | | | |
| MET: <600 | 56414 | 800/611806 | 1.019 (0.989,1.050) | 0.760 |
| MET: 600-1500 | 57505 | 862/626789 | 1.020 (0.992,1.050) |  |
| MET: ≥1500 | 19366 | 287/212679 | 1.007 (0.962,1.055) |  |

Data are hazard ratios (95% CIs). Models were adjusted for age, sex, ethnicity, BMI, SBP, LDL-C, Townsend Deprivation Index, cigarette smoking, alcohol consumption, qualification, physical activity, use of lipid-lowering medication, total energy, total sugars, sodium, red and processed meat, fruit, vegetables, saturated fatty acids, monounsaturated fatty acids, and fibre.

BMI, body mass index; LDL-C, low density lipoprotein cholesterol; CI, confidence interval; HR, hazard ratio; MET, metabolic equivalent task; SBP, systolic blood pressure.

**Supplemental Table 12.** **Associations of the risk of incident CAD by polygenic risk score (PRS)**

| **Genetic risk for CAD** | **Total No. of Participants** | **Cases/**  **Person-years** | **Crude** | **Model 1** | **Model 2** |
| --- | --- | --- | --- | --- | --- |
|  |  |  | **HR (95% CI); *P*** | **HR (95% CI); *P*** | **HR (95% CI); *P*** |
| **Low genetic risk** | 53610 | 838/581463 | 1[Ref] | 1[Ref] | 1[Ref] |
| **High genetic risk** | 53610 | 1353/578729 | 1.660 (1.523,1.809);  <0.001 | 1.586 (1.454,1.729);  <0.001 | 1.587 (1.455,1.730);  <0.001 |
|  |  |  |  |  |  |

Analyses were conducted using Cox proportional hazard models. Data are hazard ratios (95% CIs). Crude was adjusted for age, sex, and first 10 principal components of ancestry. Model 1 was adjusted as in Crude and for BMI, SBP, LDL-C, Townsend Deprivation Index, cigarette smoking, alcohol consumption, qualification, physical activity, and use of lipid-lowering medication. Model 2 was adjusted as in model 1 and for total energy, total sugars, sodium, red and processed meat, fruit, vegetables, saturated fatty acids, monounsaturated fatty acids, and fibre. *P* < 0.05 was considered statistically significant.

BMI, body mass index; CAD, coronary artery disease; CI, confidence interval; HR, hazard ratio; LDL-C, low density lipoprotein cholesterol; SBP, systolic blood pressure.

**Supplemental Table 13.** **Associations of the risk of incident PAD by polygenic risk score (PRS)**

| **Genetic risk for PAD** | **Total No. of Participants** | **Cases/**  **Person-years** | **Crude** | **Model 1** | **Model 2** |
| --- | --- | --- | --- | --- | --- |
|  |  |  | **HR (95% CI); *P*** | **HR (95% CI); *P*** | **HR (95% CI); *P*** |
| **Low genetic risk** | 53608 | 330/583780 | 1[Ref] | 1[Ref] | 1[Ref] |
| **High genetic risk** | 53612 | 386/584033 | 1.180 (1.019,1.367);  0.027 | 1.169 (1.009,1.355);  0.037 | 1.172 (1.012,1.358);  0.034 |
|  |  |  |  |  |  |

Analyses were conducted using Cox proportional hazard models. Data are hazard ratios (95% CIs). Crude was adjusted for age, sex, and first 10 principal components of ancestry. Model 1 was adjusted as in Crude and for BMI, SBP, LDL-C, Townsend Deprivation Index, cigarette smoking, alcohol consumption, qualification, physical activity, and use of lipid-lowering medication. Model 2 was adjusted as in model 1 and for total energy, total sugars, sodium, red and processed meat, fruit, vegetables, saturated fatty acids, monounsaturated fatty acids, and fibre. *P* < 0.05 was considered statistically significant.

BMI, body mass index; CI, confidence interval; HR, hazard ratio; LDL-C, low density lipoprotein cholesterol; PAD, peripheral arterial disease; SBP, systolic blood pressure.

**Supplemental Table 14.** **Associations of the risk of incident HF by polygenic risk score (PRS)**

| **Genetic risk for HF** | **Total No. of Participants** | **Cases/**  **Person-years** | **Crude** | **Model 1** | **Model 2** |
| --- | --- | --- | --- | --- | --- |
|  |  |  | **HR (95% CI); *P*** | **HR (95% CI); *P*** | **HR (95% CI); *P*** |
| **Low genetic risk** | 53610 | 740/582845 | 1[Ref] | 1[Ref] | 1[Ref] |
| **High genetic risk** | 53610 | 889/582144 | 1.203 (1.091,1.326);  <0.001 | 1.210 (1.097,1.334);  <0.001 | 1.211 (1.098,1.335);  <0.001 |
|  |  |  |  |  |  |

Analyses were conducted using Cox proportional hazard models. Data are hazard ratios (95% CIs). Crude was adjusted for age, sex, and first 10 principal components of ancestry. Model 1 was adjusted as in Crude and for BMI, SBP, LDL-C, Townsend Deprivation Index, cigarette smoking, alcohol consumption, qualification, physical activity, and use of lipid-lowering medication. Model 2 was adjusted as in model 1 and for total energy, total sugars, sodium, red and processed meat, fruit, vegetables, saturated fatty acids, monounsaturated fatty acids, and fibre. *P* < 0.05 was considered statistically significant.

BMI, body mass index; CI, confidence interval; HF, heart failure; HR, hazard ratio; LDL-C, low density lipoprotein cholesterol; SBP, systolic blood pressure.

**Supplemental Table 15.** **Sensitivity analysis 1 on the association of** **artificial sweetener intake with incident cardiovascular disease mortality, cardiovascular disease, coronary artery disease, peripheral arterial disease, stroke****, heart failure, and atrial fibrillation by excluding events that occurred within the first two years of follow-up**

|  | **Total No. of Participants** | **Cases/**  **Person-years** | **Crude** | **Model l** | **Model 2** |
| --- | --- | --- | --- | --- | --- |
|  |  |  | **HR (95% CI); *P*** | **HR (95% CI); *P*** | **HR (95% CI); *P*** |
| CVD mortality | 132507 | 899/1456632 | 1.043 (1.015,1.071);  0.003 | 1.020 (0.992,1.049);  0.164 | 1.018 (0.990,1.047);  0.201 |
|  |  |  |  |  |  |
| Overall CVD | 125550 | 31045/1243071 | 1.035 (1.029,1.040);  <0.001 | 1.015 (1.010,1.021);  <0.001 | 1.014 (1.009,1.020);  <0.001 |
|  |  |  |  |  |  |
| CAD | 132206 | 2344/1444042 | 1.042 (1.024,1.060);  <0.001 | 1.019 (1.001,1.037);  0.043 | 1.018 (1.000,1.036);  0.054 |
|  |  |  |  |  |  |
| PAD | 132450 | 793/1453690 | 1.076 (1.050,1.102);  <0.001 | 1.041 (1.014,1.067);  0.002 | 1.039 (1.013,1.066);  0.003 |
|  |  |  |  |  |  |
| Stroke | 132317 | 1694/1449451 | 1.012 (0.988,1.036);  0.334 | 1.001 (0.977,1.025);  0.958 | 0.999 (0.976,1.024);  0.965 |
| HF | 132380 | 1806/1450323 | 1.045 (1.026,1.065);  <0.001 | 1.019 (1.000,1.039);  0.055 | 1.018 (0.998,1.038);  0.072 |
| AF | 131885 | 5228/1431423 | 1.013 (1.000,1.027);  0.055 | 1.001 (0.988,1.015);  0.871 | 1.000 (0.987,1.014);  0.985 |
|  |  |  |  |  |  |

Data are hazard ratios (95% CIs). Crude was adjusted for age, sex, ethnicity. Model 1 was adjusted as in Crude and for BMI, SBP, LDL-C, Townsend Deprivation Index, cigarette smoking, alcohol consumption, qualification, physical activity, use of lipid-lowering medication. Model 2 was adjusted as in model 1 and for total energy, total sugars, sodium, red and processed meat, fruit, vegetables, saturated fatty acids, monounsaturated fatty acids, and fibre. *P* < 0.05 was considered statistically significant.

AF, atrial fibrillation; BMI, body mass index; CAD, coronary artery disease; CVD, cardiovascular disease; CI, confidence interval; HF, heart failure; HR, hazard ratio; LDL-C, low density lipoprotein cholesterol; PAD, peripheral arterial disease; SBP, systolic blood pressure.

**Supplemental Table 16. Sensitivity analysis 2 on the association of artificial sweetener intake with incident cardiovascular disease mortality, cardiovascular disease, coronary artery disease, peripheral arterial disease, stroke, heart failure, and atrial fibrillation among participants with two or more** **dietary questionnaires based on a 24-hour dietary recall**

|  | **Total No. of Participants** | **Cases/**  **Person-years** | **Crude** | **Model l** | **Model 2** |
| --- | --- | --- | --- | --- | --- |
|  |  |  | **HR (95% CI); *P*** | **HR (95% CI); *P*** | **HR (95% CI); *P*** |
| CVD mortality | 76258 | 484/798696 | 1.032 (0.989,1.077);  0.145 | 1.011 (0.967,1.056);  0.636 | 1.007 (0.963,1.052);  0.767 |
| Overall CVD | 76258 | 20312/694660 | 1.034 (1.027,1.041);  <0.001 | 1.013 (1.006,1.020);  <0.001 | 1.013 (1.005,1.020);  0.001 |
| CAD | 76258 | 1313/792985 | 1.050 (1.026,1.075);  <0.001 | 1.031 (1.006,1.056);  0.016 | 1.029 (1.004,1.055);  0.021 |
| PAD | 76258 | 414/797357 | 1.092 (1.055,1.131);  <0.001 | 1.053 (1.015,1.091);  0.005 | 1.046 (1.008,1.085);  0.016 |
| Stroke | 76258 | 965/795294 | 0.993 (0.957,1.030);  0.695 | 0.983 (0.946,1.020);  0.363 | 0.981 (0.945,1.019);  0.326 |
| HF | 76258 | 989/795571 | 1.048 (1.019,1.077);  0.001 | 1.020 (0.991,1.049);  0.180 | 1.018 (0.989,1.048);  0.227 |
| AF | 76258 | 3176/785916 | 0.995 (0.976,1.015);  0.639 | 0.984 (0.964,1.004);  0.109 | 0.983 (0.963,1.003);  0.099 |
|  |  |  |  |  |  |

Data are hazard ratios (95% CIs). Crude was adjusted for age, sex, ethnicity. Model 1 was adjusted as in Crude and for BMI, SBP, LDL-C, Townsend Deprivation Index, cigarette smoking, alcohol consumption, qualification, physical activity, use of lipid-lowering medication. Model 2 was adjusted as in model 1 and for total energy, total sugars, sodium, red and processed meat, fruit, vegetables, saturated fatty acids, monounsaturated fatty acids, and fibre. *P* < 0.05 was considered statistically significant.

AF, atrial fibrillation; BMI, body mass index; CAD, coronary artery disease; CVD, cardiovascular disease; CI, confidence interval; HF, heart failure; HR, hazard ratio; LDL-C, low density lipoprotein cholesterol; PAD, peripheral arterial disease; SBP, systolic blood pressure.

**Supplemental Table 17. Sensitivity analysis 3 on** **the association of artificial sweetener intake with incident cardiovascular disease mortality, cardiovascular disease, coronary artery disease, peripheral arterial disease, stroke, heart failure, and atrial fibrillation among participants with complete covariate data**

|  | **Total No. of Participants** | **Cases/**  **Person-years** | **Crude** | **Model l** | **Model 2** |
| --- | --- | --- | --- | --- | --- |
|  |  |  | **HR (95% CI); *P*** | **HR (95% CI); *P*** | **HR (95% CI); *P*** |
| CVD mortality | 86215 | 619/943131 | 1.012 (0.973,1.053); | 0.992 (0.953,1.033); | 0.990 (0.950,1.031); |
|  |  |  | 0.553 | 0.704 | 0.615 |
| Overall CVD | 86215 | 24796/809396 | 1.037 (1.031,1.043); | 1.019 (1.012,1.025); | 1.018 (1.011,1.024); |
|  |  |  | <0.001 | <0.001 | <0.001 |
| CAD | 86215 | 1720/935169 | 1.043 (1.021,1.064); | 1.021 (1.000,1.043); | 1.020 (0.998,1.042); |
|  |  |  | <0.001 | 0.056 | 0.069 |
| PAD | 86215 | 559/941321 | 1.071 (1.038,1.105); | 1.036 (1.003,1.071); | 1.035 (1.002,1.069); |
|  |  |  | <0.001 | 0.031 | 0.040 |
| Stroke | 86215 | 1233/938479 | 1.005 (0.975,1.035); | 0.994 (0.965,1.025); | 0.993 (0.963,1.023); |
|  |  |  | 0.759 | 0.712 | 0.649 |
| HF | 86215 | 1272/939022 | 1.027 (1.001,1.053); | 1.003 (0.977,1.030); | 1.002 (0.976,1.029); |
|  |  |  | 0.046 | 0.829 | 0.898 |
| AF | 86215 | 3810/927180 | 1.015 (0.999,1.031);  0.071 | 1.004 (0.988,1.021);  0.590 | 1.003 (0.987,1.020);  0.698 |
|  |  |  |  |  |  |

Data are hazard ratios (95% CIs). Crude was adjusted for age, sex, ethnicity. Model 1 was adjusted as in Crude and for BMI, SBP, LDL-C, Townsend Deprivation Index, cigarette smoking, alcohol consumption, qualification, physical activity, use of lipid-lowering medication. Model 2 was adjusted as in model 1 and for total energy, total sugars, sodium, red and processed meat, fruit, vegetables, saturated fatty acids, monounsaturated fatty acids, and fibre. *P* < 0.05 was considered statistically significant.

AF, atrial fibrillation; BMI, body mass index; CAD, coronary artery disease; CVD, cardiovascular disease; CI, confidence interval; HF, heart failure; HR, hazard ratio; LDL-C, low density lipoprotein cholesterol; PAD, peripheral arterial disease; SBP, systolic blood pressure.

**Supplementary Table 18. Sensitivity analysis 4 on the association of artificial sweetener intake with incident cardiovascular disease mortality, cardiovascular disease, coronary artery disease, peripheral arterial disease, stroke, heart failure, and atrial fibrillation by** **not excluding participants with baseline diabetes**

|  | **Total No. of Participants** | **Cases/**  **Person-years** | **Crude** | **Model l** | **Model 2** |
| --- | --- | --- | --- | --- | --- |
|  |  |  | **HR (95% CI); *P*** | **HR (95% CI); *P*** | **HR (95% CI); *P*** |
| CVD mortality | 136229 | 1038/1489434 | 1.037 (1.011,1.064);  0.005 | 1.014 (0.988,1.041);  0.289 | 1.013 (0.986,1.040);  0.346 |
| Overall CVD | 136229 | 39505/1275872 | 1.034 (1.030,1.039);  <0.001 | 1.013 (1.009,1.018);  <0.001 | 1.013 (1.008,1.017);  <0.001 |
| CAD | 136229 | 2771/1476637 | 1.046 (1.030,1.062);  <0.001 | 1.021 (1.005,1.037);  0.009 | 1.020 (1.004,1.037);  0.012 |
| PAD | 136229 | 888/1486466 | 1.068 (1.043,1.093);  <0.001 | 1.031 (1.006,1.057);  0.016 | 1.029 (1.004,1.055);  0.022 |
| Stroke | 136229 | 1977/1482200 | 1.008 (0.986,1.030);  0.486 | 0.995 (0.973,1.018);  0.678 | 0.995 (0.972,1.017);  0.633 |
| HF | 136229 | 2035/1482959 | 1.049 (1.031,1.067);  <0.001 | 1.022 (1.004,1.040);  0.015 | 1.021 (1.003,1.039);  0.021 |
| AF | 136229 | 6055/1464282 | 1.012 (1.000,1.025);  0.047 | 1.001 (0.989,1.013);  0.873 | 1.000 (0.988,1.012);  0.989 |
|  |  |  |  |  |  |

Data are hazard ratios (95% CIs). Crude was adjusted for age, sex, ethnicity. Model 1 was adjusted as in Crude and for BMI, SBP, LDL-C, Townsend Deprivation Index, cigarette smoking, alcohol consumption, qualification, physical activity, use of lipid-lowering medication, and baseline diabetes. Model 2 was adjusted as in model 1 and for total energy, total sugars, sodium, red and processed meat, fruit, vegetables, saturated fatty acids, monounsaturated fatty acids, and fibre. *P* < 0.05 was considered statistically significant.

AF, atrial fibrillation; BMI, body mass index; CAD, coronary artery disease; CVD, cardiovascular disease; CI, confidence interval; HF, heart failure; HR, hazard ratio; LDL-C, low density lipoprotein cholesterol; PAD, peripheral arterial disease; SBP, systolic blood pressure.

**Supplemental Table 19. Sensitivity analysis 5 on the association of artificial sweetener intake with incident cardiovascular disease mortality, cardiovascular disease, coronary artery disease, peripheral arterial disease, stroke, heart failure, and atrial fibrillation** **by** **adjusting the consumption clusters**

|  | **Total No. of Participants** | **Cases/**  **Person-years** | **Main analysis** | **Additional Model 1** | **Additional Model 2** | **Additional Model 3** |
| --- | --- | --- | --- | --- | --- | --- |
|  |  |  | **HR (95% CI);** P | **HR (95% CI);** P | **HR (95% CI);** P | **HR (95% CI);** P |
| CVD mortality | 133285 | 991/1457448 | 1.014 (0.987,1.043);  0.305 | 1.015 (0.988,1.044);  0.271 | 1.016 (0.988,1.044);  0.268 | 1.015 (0.988,1.043);  0.286 |
| Overall CVD | 133285 | 38260/1250882 | 1.012 (1.008,1.017);  <0.001 | 1.013 (1.008,1.018);  <0.001 | 1.013 (1.008,1.018);  <0.001 | 1.013 (1.008,1.018);  <0.001 |
| CAD | 133285 | 2655/1445201 | 1.018 (1.001,1.035);  0.034 | 1.019 (1.002,1.036);  0.026 | 1.019 (1.002,1.036);  0.027 | 1.018 (1.001,1.035);  0.036 |
| PAD | 133285 | 857/1454570 | 1.035 (1.010,1.061);  0.006 | 1.037 (1.012,1.063);  0.004 | 1.034 (1.008,1.060);  0.009 | 1.036 (1.010,1.062);  0.006 |
| Stroke | 133285 | 1912/1450456 | 0.998 (0.975,1.021);  0.868 | 0.998 (0.976,1.021);  0.880 | 0.997 (0.975,1.021);  0.818 | 0.998 (0.975,1.021);  0.842 |
| HF | 133285 | 1949/1451274 | 1.018 (0.999,1.038);  0.061 | 1.018 (0.999,1.038);  0.060 | 1.019 (1.000,1.039);  0.051 | 1.018 (0.999,1.037);  0.063 |
| AF | 133285 | 5889/1432914 | 0.998 (0.986,1.011);  0.805 | 0.998 (0.986,1.011);  0.789 | 0.998 (0.986,1.011);  0.808 | 0.999 (0.986,1.012);  0.836 |
|  |  |  |  |  |  |  |

Analyses were conducted using Cox proportional hazard models. Data are hazard ratios (95% CIs). Main analysis was adjusted for age, sex, ethnicity, BMI, SBP, LDL-C, Townsend Deprivation Index, cigarette smoking, alcohol consumption, qualification, physical activity, use of lipid-lowering medication, total energy, total sugars, sodium, red and processed meat, fruit, vegetables, saturated fatty acids, monounsaturated fatty acids, and fibre. P < 0.05 was considered statistically significant.

Additional Model 1, main analysis, adjusted for added sugars and preserves, not adjusted for total sugars.

Additional Model 2, main analysis, additionally adjusted for coffee consumption, tea consumption, and cereal consumption.

Additional Model 3, main analysis, adjusted for “ideal diet” (yes or no), not adjusted for red and processed meat, fruit, and vegetables.

AF, atrial fibrillation; BMI, body mass index; CAD, coronary artery disease; CVD, cardiovascular disease; CI, confidence interval; HF, heart failure; HR, hazard ratio; LDL-C, low density lipoprotein cholesterol; PAD, peripheral arterial disease; SBP, systolic blood pressure.

**Supplemental Figure 1.** **Restricted cubic spline for the association between artificial sweetener intake and the risk of incident CVD and mortality.** Models were adjusted for age, sex, ethnicity, BMI, SBP, LDL-C, Townsend Deprivation Index, cigarette smoking, alcohol consumption, qualification, physical activity, use of lipid-lowering medication, total energy, total sugars, sodium, red and processed meat, fruit, vegetables, saturated fatty acids, monounsaturated fatty acids, and fibre. AF, atrial fibrillation; BMI, body mass index; CAD, coronary artery disease; CVD, cardiovascular disease; CI, confidence interval; HF, heart failure; HR, hazard ratio; LDL-C, low density lipoprotein cholesterol; PAD, peripheral arterial disease; SBP, systolic blood pressure


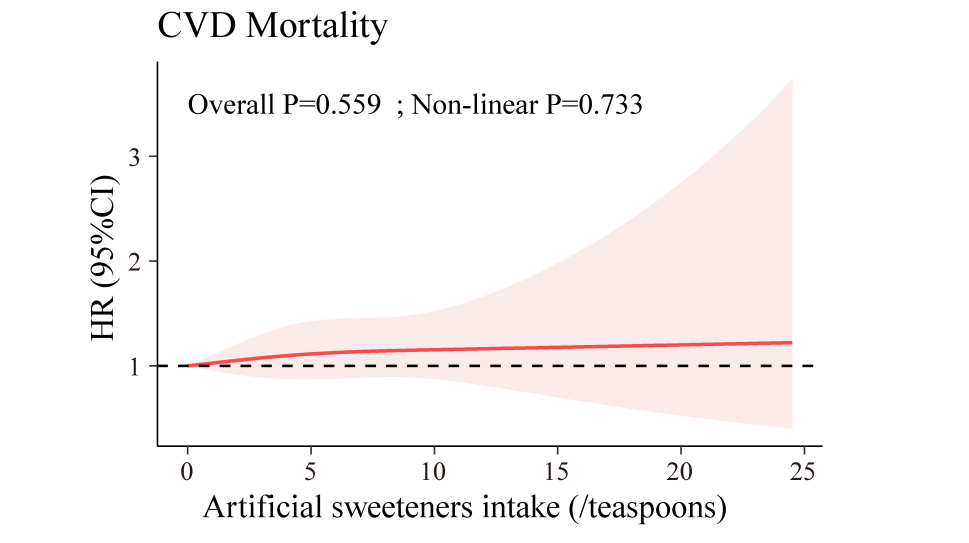


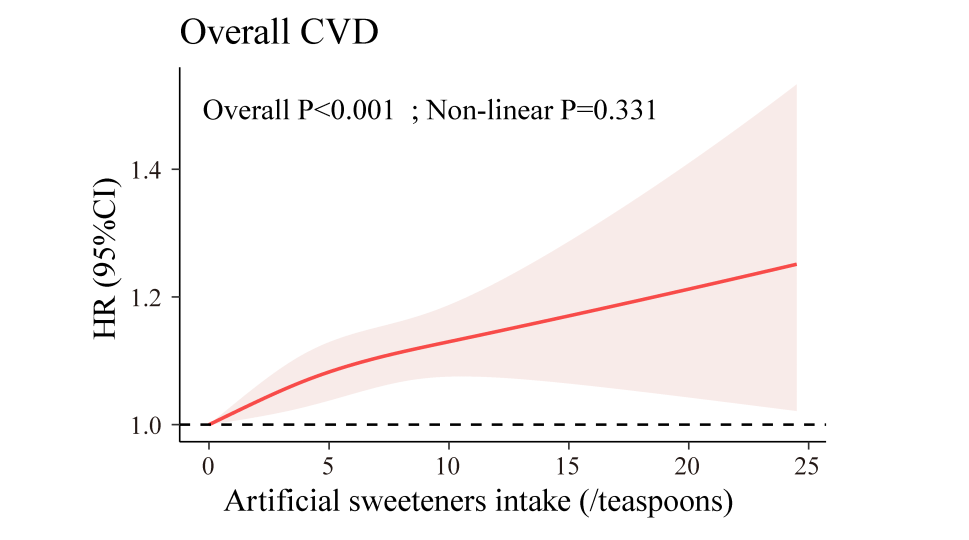


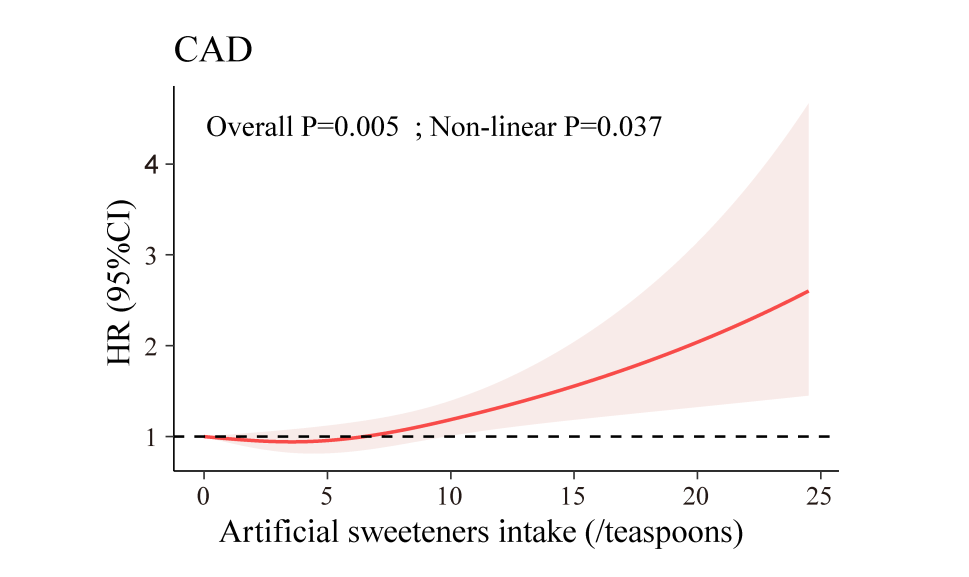


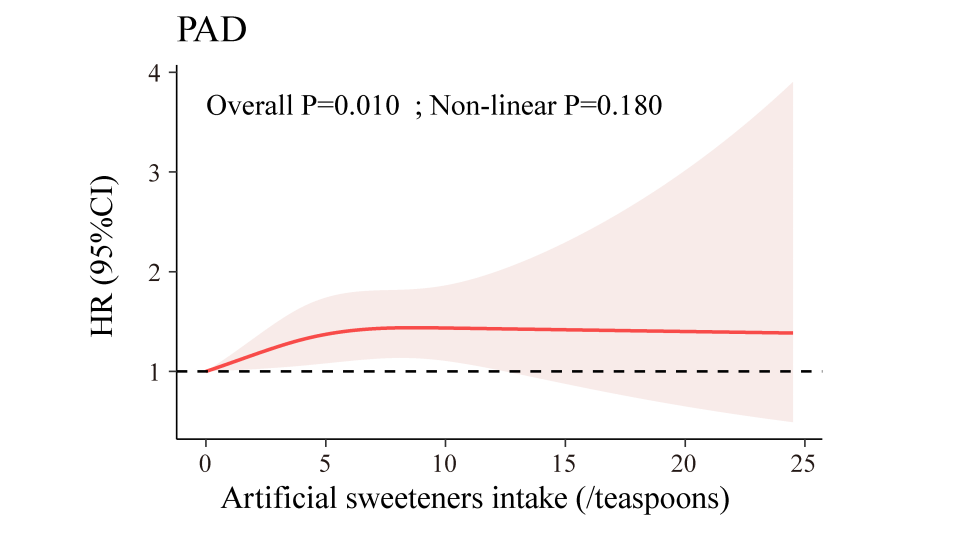


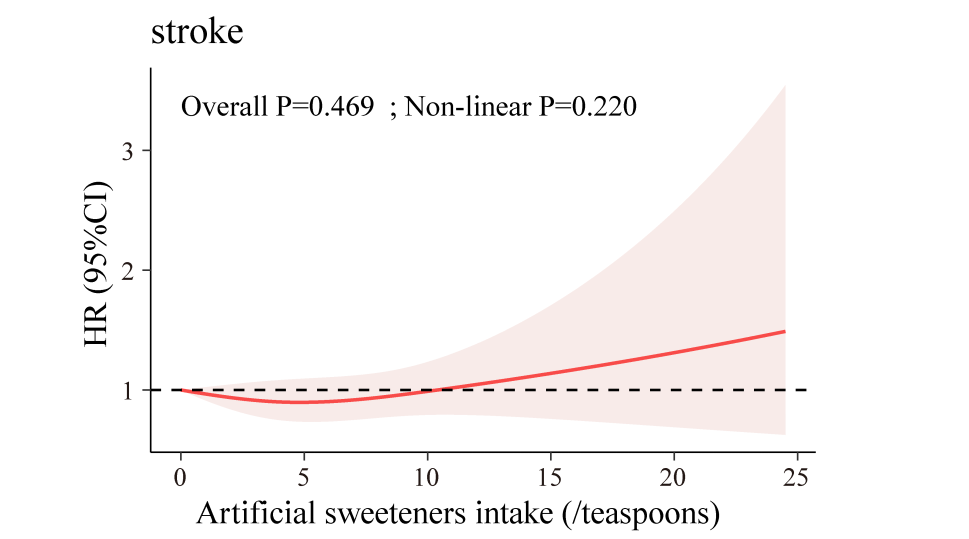


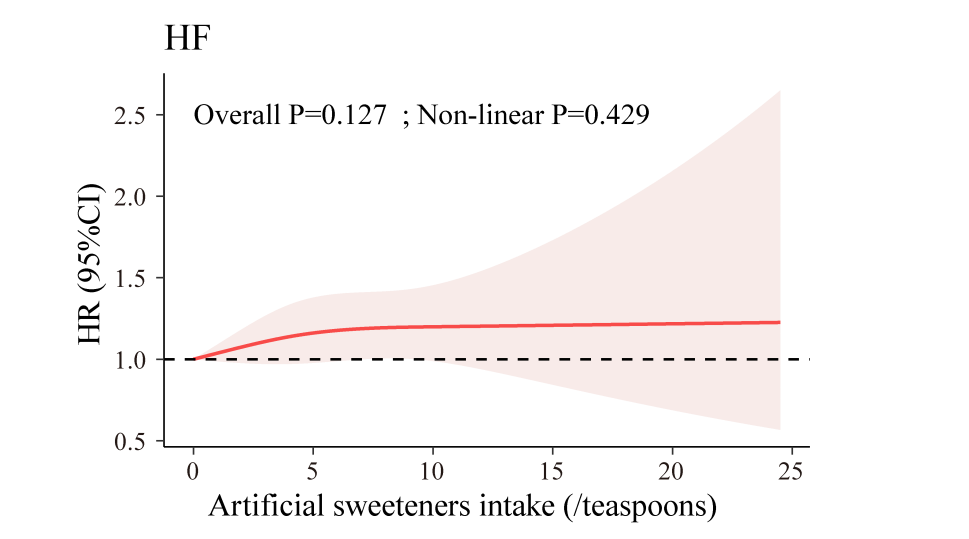


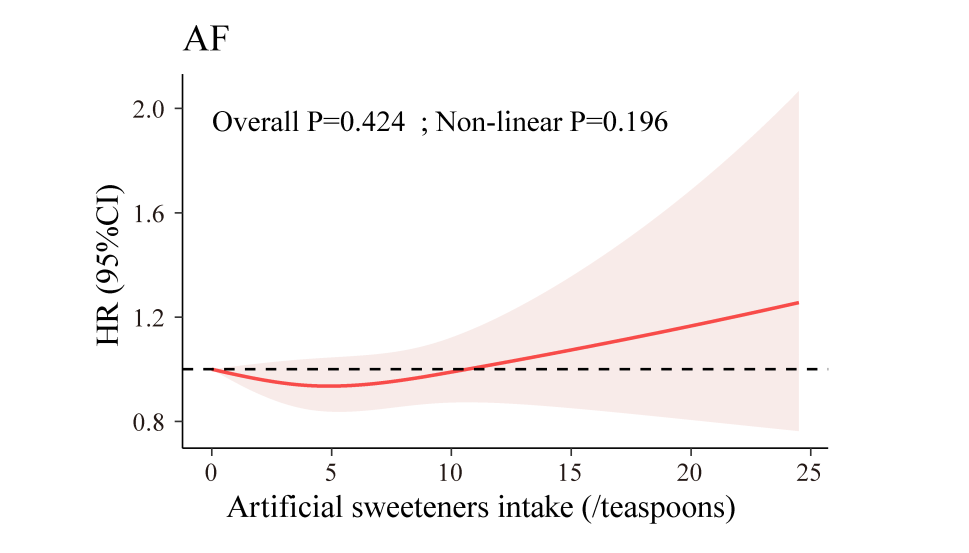

Supplement: Supplementary file 1 — Supplementary Material 1 [file 12933_2024_2333_MOESM1_ESM.docx]
